# Affinity‐Based Protein Profiling Revealed that HIGD1A is a Direct Target Protein of Aristolochic Acids

**DOI:** 10.1002/advs.202513117

**Published:** 2025-11-05

**Authors:** Yin Gong, Shanshan Zhang, Anying Wei, Xiang Ji, Xiaodan Chong, Jinfeng Cen, Xuan Zhao, Zimeng Luo, Zhipeng Pei, Guanchao Mao, Xinkang Zhang, Mingxue Sun, Zhiguo Sun, Zifei Yin, Zhengrong Zou, Wen‐Qi Meng

**Affiliations:** ^1^ Faculty of Naval Medicine Naval Military Medical University Shanghai China; ^2^ College of Life Sciences Jiangxi Normal University Nanchang 330022 China; ^3^ School of Traditional Chinese Medicine Naval Medical University Shanghai 200433 China; ^4^ Clinical Cancer Institute Translational Medicine Center Faculty of Pharmacy Naval Medical University Shanghai 200433 China; ^5^ Department of Pharmaceutical Science Faculty of Pharmacy Naval Medical University Shanghai 200433 China

**Keywords:** aristolochic acids, affinity‐based probe, HIGD1A, mitochondrial dysfunction

## Abstract

Exposure to aristolochic acids (AA) via the ingestion of AA‐containing medicine or food is a significant risk factor for severe nephropathy. Despite decades of research, the direct molecular targets underlying AA toxicity remain elusive. In this study, a map of the AA‐binding protein atlas in kidney tissues is constructed via the design and synthesis of an AA‐based affinity probe. Among the AA‐binding proteins, HIGD1A is identified as a high‐affinity AA target (SPR: 59.6 nm; ITC: 195 nm) with significant thermostability changes upon AA binding. Furthermore, AA disrupted the HIGD1A‐TFAM interaction, triggering TFAM degradation via the autophagy‐lysosome pathway. This led to the accumulation of cytosolic mitochondrial DNA. This study also suggested that HIGD1A also contributed to AA‐induced inflammation by increasing the cytosolic levels of oxidized mitochondrial DNA to activate the MAPK and NF‐κB pathways. This study not only reveals an important target protein of AA but also provides novel inspiration and ideas for toxicology and chemical biology research.

## Graphical abstract

Using an affinity‐based probe, this study reveals that aristolochic acid directly binds to HIGD1A with high affinity in the kidney. This binding disrupts the HIGD1A‐TFAM interaction, which in turn triggers TFAM degradation, cytosolic mitochondrial DNA release, and the activation of inflammatory pathways.

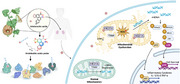

## 1 Introduction

Aristolochic acids (AA) are nitrophenanthrene carboxylic acids that are derived mainly from herbal medical plants, including *Asarum* and *Aristolochia*.^[^ ^1^, ^2^ ^]^ Medicines that contain AA have been used for decades to treat various diseases, including urinary tract infection, hepatitis, edema, and eczema.^[^ ^3^ ^]^ In the 1990s, an unusual renal disease that was characterized by rapidly progressive tubulointerstitial nephritis was observed among young female patients in Belgium, and AA, which were components of medicines taken to promote weight loss, were identified as the causative agent of this pathology.^[^ ^4^, ^5^, ^6^ ^]^ The pathological hallmark of AA‐induced nephropathy is extensive interstitial fibrosis associated with ischemic glomerular and tubular atrophy.^[^ ^7^, ^8^, ^9^ ^]^ In 2002, the International Agency for Research on Cancer (IARC) classified AA as Group I carcinogens in humans, and AA were shown to exert their effect via a genotoxic mechanism.^[^ ^10^, ^11^ ^]^ In‐depth studies on both the possible therapeutic effects and toxic effects of AA are warranted.

Numerous studies on the mechanisms underlying AA‐induced nephrotoxicity have been conducted in the past 30 years.^[^ ^3^, ^12^, ^13^, ^14^, ^15^ ^]^ AA can be reduced to reactive cyclic acylnitrenium ions and subsequently bind to the exocyclic amino groups of dA, dG, and dC, forming covalent DNA adducts in both mitochondria and nuclei.^[^ ^9^, ^16^, ^17^ ^]^ Additionally, AA cause mitochondrial dysfunction, oxidative stress, and apoptosis via the NLRP3, PI3K/Akt, and MEK/ERK signaling pathways.^[^ ^12^, ^18^ ^]^ Some studies have demonstrated that inflammation and fibrosis are the pathogenic characteristics of AA‐induced nephrotoxicity, which is caused by the activation of TGF‐dependent and c‐Jun N‐terminal kinase (JNK)/MAP kinase‐dependent signaling pathways and the C3a complement system.^[^ ^19^, ^20^ ^]^ Although our knowledge of the mechanism underlying AA‐induced nephrotoxicity has greatly improved over the past 30 years, most related research has focused on the DNA adducts of AA and several classical proteins related to oxidative stress or inflammation.

In this study, we aimed to perform a comprehensive characterization of AA‐interacting proteins in mouse kidneys. Our approach was inspired by previous efforts to map drugs and endogenous metabolites, e.g., metformin and glucose, by using affinity‐based protein profiling (A*f*BPP).^[^ ^21^, ^22^ ^]^ We first confirmed the acute toxic effects of AA in mice and living cells. We then designed and synthesized an AA affinity‐based probe and applied the probe to profile all AA‐interacting proteins directly in mouse kidneys via A*f*BPP. With this strategy, we identified 32 high‐confidence AA‐interacting proteins that were functionally enriched in mitochondrion, lysosome, and lipase inhibitor activity. On the basis of the results of affinity‐based protein profiling, we further found that Hypoxia‐induced gene domain protein‐1a (HIGD1A) had a high affinity for AA. And AA disrupts the HIGD1A‐mitochondrial transcription factor A (TFAM, a key regulator of mtDNA) interaction, triggering TFAM degradation via autophagy‐lysosomal pathways. This loss of TFAM stability explains subsequent accumulation of cytosolic mtDNA, which may activate MAPK/NF‐κB inflammation cascades. The overexpression of HIGD1A significantly inhibited AA‐induced mitochondrial dysfunction and inflammation. Our work not only provides the comprehensive AA‐interactome atlas but also identifies HIGD1A‐TFAM complex stabilization as a novel therapeutic target against AA nephrotoxicity.

## 2 Results

### 2.1 Acute Toxic Effects of Aristolochic Acids on Cells and Mice

**Figure 1:**
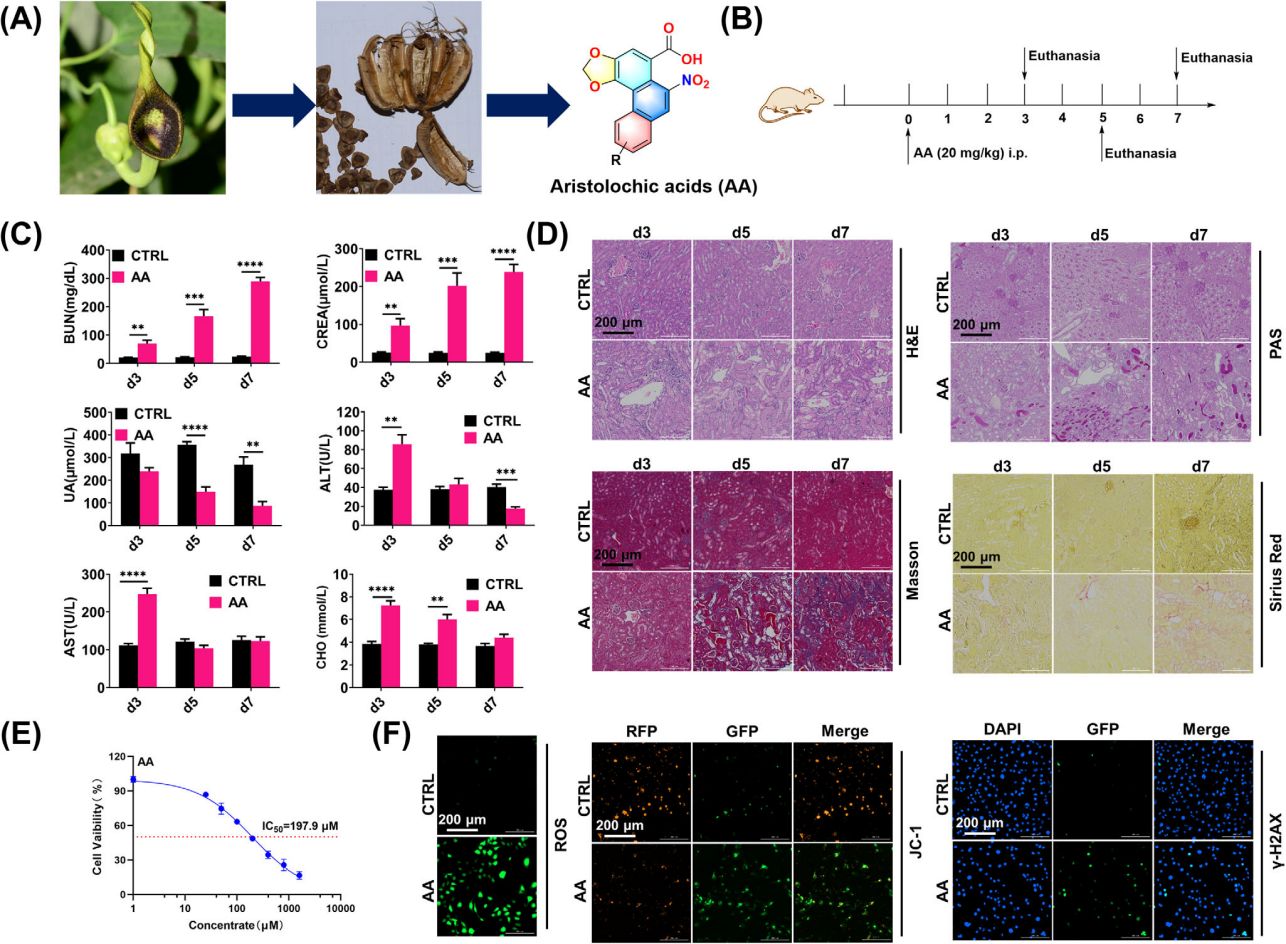
AA causes mouse kidney injury and HK‐2 cell damage. A) *Aristolochia debilis* Sieb. et Zucc. and the chemical structure of AA. B) Schematic of the AA‐induced kidney injury animal experiment. C) Effects of AA on the levels of serum indices in the mice. D) Kidney sections subjected to H&E, PAS, Masson and Sirius Red staining. E) HK‐2 cell viability after exposure to different concentrations of AA. F,G) Effects of AA (100 µm) on ROS production, mitochondrial depolarization, and γ‐H2A expression in HK‐2 cells. Student's *t*‐test is used to compare two groups of data affected by a single variable. For the comparison of multiple groups of data, all data are presented as mean ± standard deviation (*n* = 3). Differences were considered statistically significant at *p < 0.05, **p < 0.01, ***p < 0.001, and ****p < 0.0001.

We first evaluated the acute toxic effects of AA on mice, especially with respect to kidney and liver injuries. The animal experiment was designed and carried out as shown in **Figure** **1**A. The AA‐induced liver injury was transient, but the AA I‐induced nephrotoxicity was significant and continued to progress (Figure 1B,C). The kidney‐related indices in mouse blood, including UA, CREA, and BUN, were clearly changed after AA exposure for 5 or 7 days, whereas most of the other blood indices were not significantly different on days 5 or 7 (Figures 1B; S1, Supporting Information). The results of the histopathology examinations were consistent with the results of blood index measurements (Figures 1C; S2, Supporting Information). After AA exposure, the glomerulus showed no significant lesions, whereas the renal tubular epithelial cells exhibited severe degeneration and necrosis, with disruption and partial exposure of the sarcolemma. Edema in the renal interstitium and inflammatory cell infiltration was also observed. We also investigated the toxicity of AA in human kidney 2 (HK‐2) cells. The IC_50_ of AA was measured via a CCK8 assay, and this value was 197.9 µm at 24 h. As shown in Figures 1E–G and S3 (Supporting Information), AA exposure caused the overproduction of ROS, a reduction in the mitochondrial membrane potential (MMP), and an increase in the expression of γ‐H2A.

### 2.2 Synthesis and Evaluation of the Toxicity of an AA‐Based Affinity Probe

**Figure 2:**
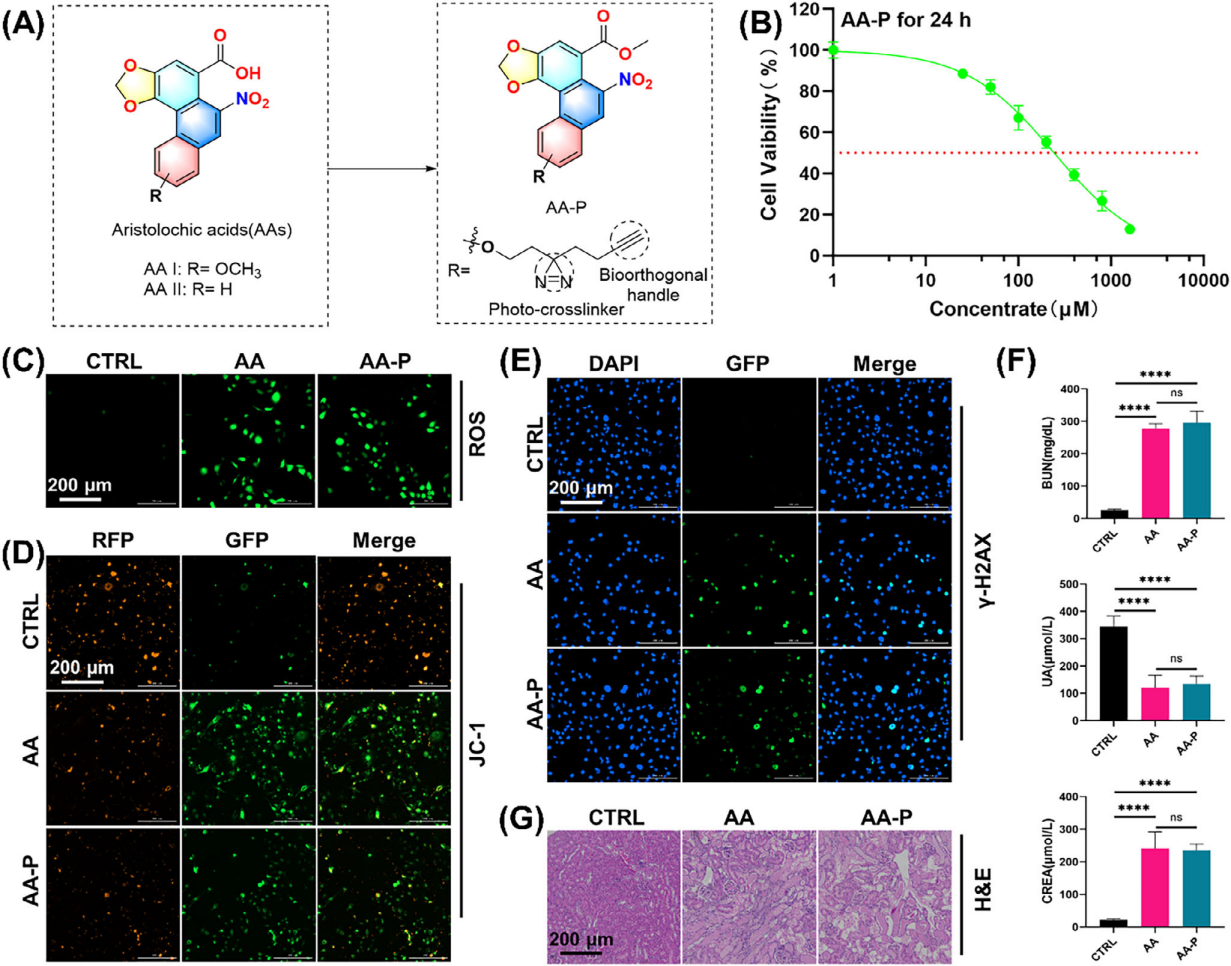
Synthesis of the AA probe AA‐P and evaluation of its toxicity. A) Chemical structures of AA and AA‐P. B) HK‐2 cell viability after exposure to different concentrations of AA‐P. C–E) Effects of AA and AA‐P on ROS production, mitochondrial depolarization, and γ‐H2A expression in HK‐2 cells. F) Effects of AA and AA‐P on the serum levels of BUN, UA and CREA in mice. G) Representative images of H&E‐stained kidneys from AA‐ and AA‐P‐treated mice. Student's *t*‐test is used to compare two groups of data affected by a single variable. For the comparison of multiple groups of data, one‐way ANOVA is adopted, and Dunnett's multiple comparisons test is used for post hoc analysis. All data are presented as mean ± standard deviation (*n* = 3). Differences were considered statistically significant at *p < 0.05, **p < 0.01, ***p < 0.001, ****p < 0.0001, and ns not significant.

To identify the direct target proteins of AA, an AA affinity‐based probe (AA‐P) was designed and synthesized with the photocrosslinker diazirine and a biorthogonal handle alkyne (**Figure** **2**A). The photocrosslinker diazirine formed a covalent bond between the probe and the target protein upon ultraviolet (UV) irradiation, and the biorthogonal handle was subsequently linked to a fluorescent dye or biotin. The AA‐P probe was characterized by ^1^H NMR, ^13^C NMR, and HPLC (Figures S4–S6, Supporting Information). After AA‐P was generated, we further examined the toxicity of AA‐P in cells and mice. The cytotoxicity of the probe was assessed in live HK‐2 cells via a CCK‐8 assay. The probe and unmodified AA had similar effects on cell proliferation (Figure 2B). We also found that both AA and AA‐P triggered excessive ROS production in HK‐2 cells, and the difference between these two conditions was not significant (Figures 2C; S6, Supporting Information). Similar to unmodified AA, AA‐P also increased γ‐H2AX expression and reduced the MMP (Figures 2D,E; S7, Supporting Information). We also further examined the toxicity of AA‐P in mice. H&E staining and blood index measurement revealed that, similar to the AA‐treated mice, the AA‐P‐treated mice exhibited severe kidney injury (Figure 2G,F). These results indicated that AA‐P has toxic effects that are similar to those of AA on both cells and mice.

### 2.3 Fluorescence Labeling of AA‐P in Cells and Mouse Kidneys

**Figure 3:**
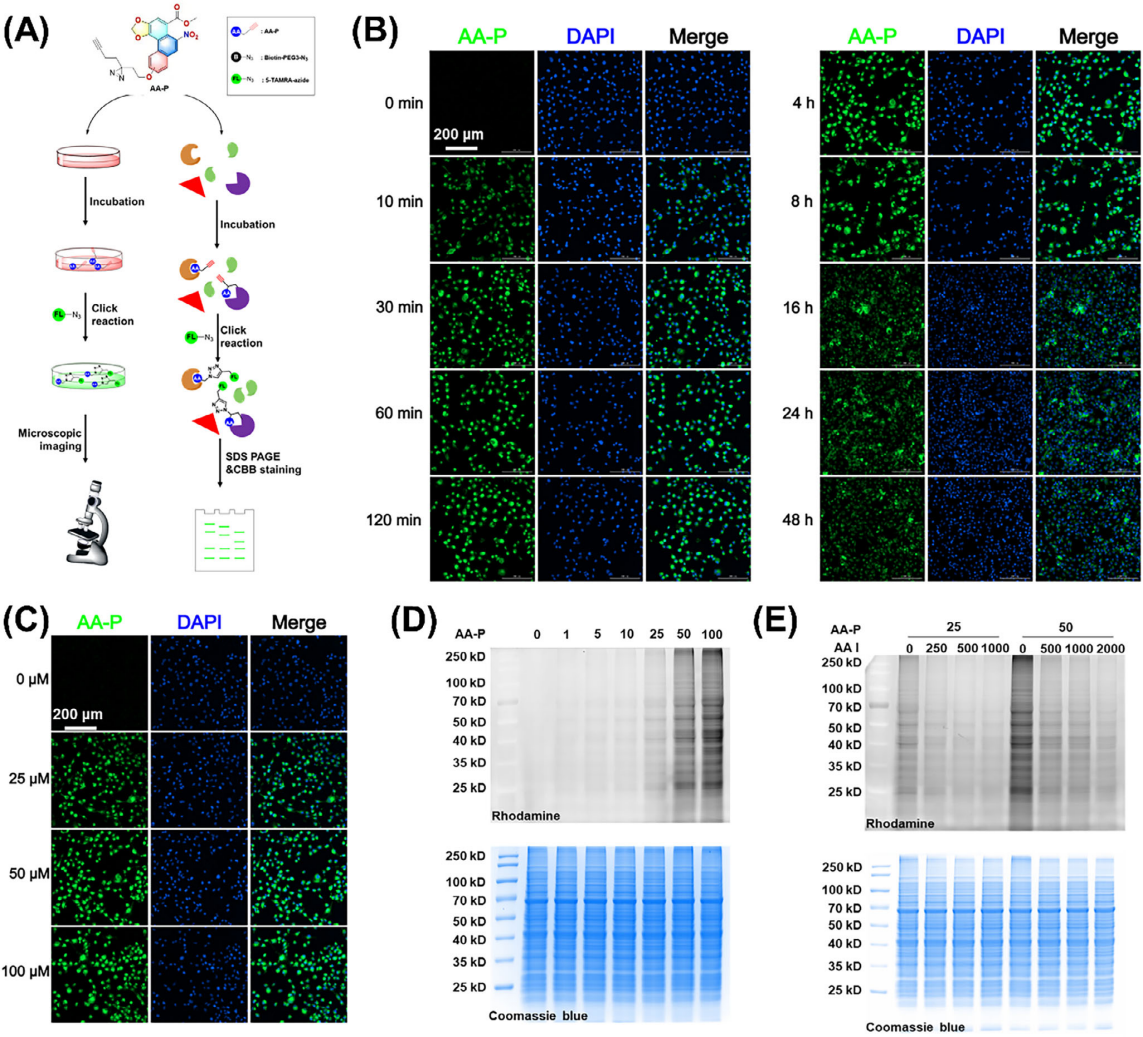
Fluorescence labeling of kidney proteins by AA‐P in living cells and mice. A) Overall experimental design for AA‐P fluorescence labeling. B) HK‐2 cells were incubated with AA‐P (50 µm) for different durations. C) HK‐2 cells were incubated with different concentrations of AA‐P (0, 25, 50, and 100 µm) for 2 h. D) Mouse kidney protein labeling with AA‐P in a dose‐dependent manner was observed via SDS‒PAGE. E) AA blocked the labeling of mouse kidney proteins by 25 and 50 µm AA‐P as shown by an SDS‒PAGE gel.

We further explored whether AA‐P could undergo effective photoaffinity labeling in cells and mouse kidneys (**Figure** **3**A). We first incubated live HK‐2 cells with the AA‐P probe (50 µm) for different durations and then washed the cells with PBS. The cells were subsequently fixed and subjected to a click reaction with 5‐TAMRA‐azide. As shown in Figure 3B, the fluorescence intensity in HK‐2 cells reached the maximum value within 2 h. Then, the fluorescence intensity decreased from 4 to 48 h. Figure 3C shows that AA‐P increased the fluorescence intensity in a concentration‐dependent manner. Then, the photoaffinity labeling ability of AA‐P in mouse kidneys was explored via SDS‒PAGE. Mouse kidneys were homogenized, and the proteins were extracted. The kidney proteins were incubated with different concentrations of AA (1, 5, 10, 25, 50, and 100 µm). AA‐P labeled kidney proteins in a dose‐dependent manner (Figure 3D). According to the fluorescence intensity, 25 and 50 µm AA‐P were chosen for the competitive labeling experiments. As shown in Figure 3E, the interaction between AA‐P and target proteins was inhibited by AA. Taken together, these findings indicated that AA‐P can be used as an AA affinity‐based probe for the fluorescence labeling of living cells and mouse kidneys.

### 2.4 Identification of AA Targets via A*f*BPP‐Based Chemical Proteomics

**Figure 4:**
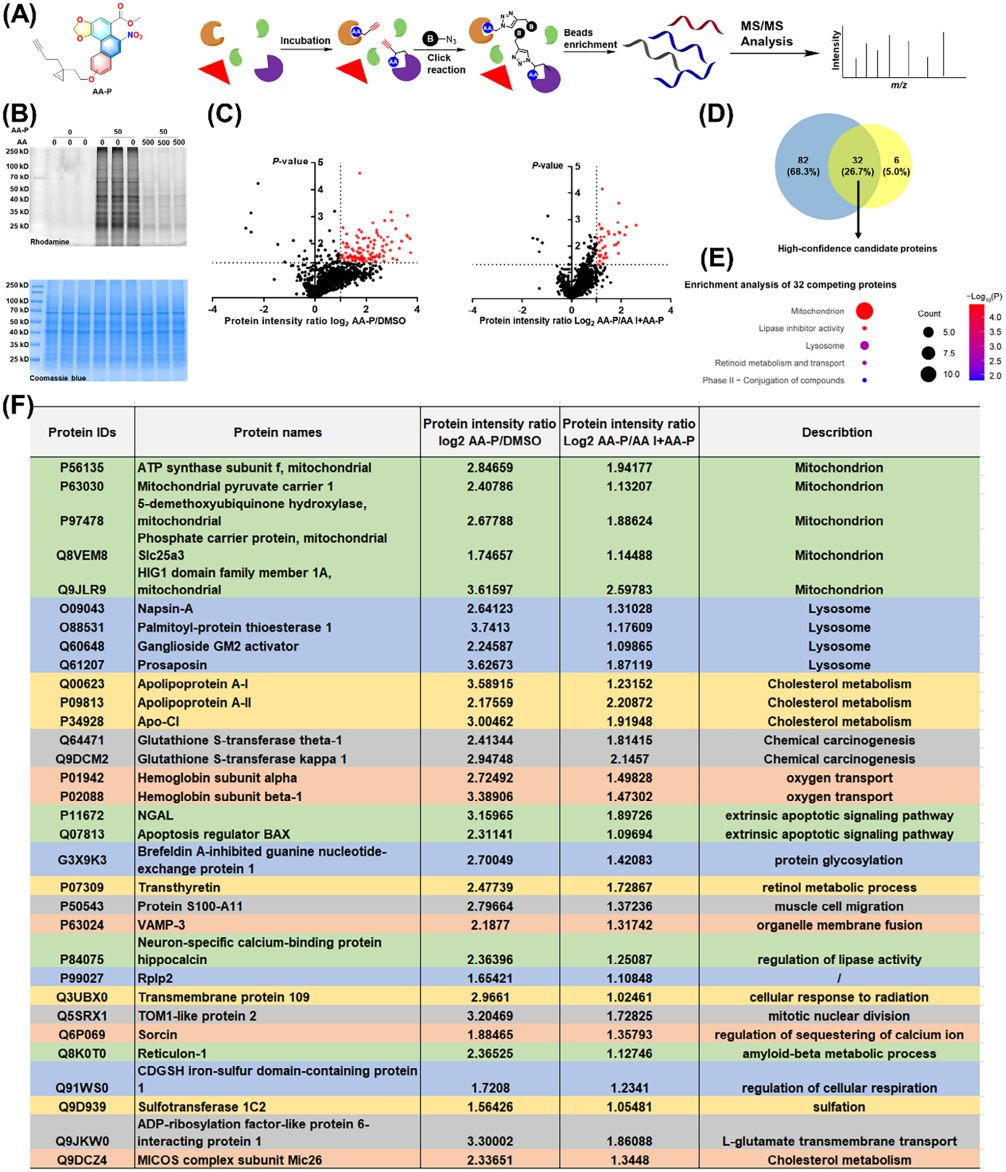
MS‐based profiling of AA‐interacting proteins in mouse kidneys by A*f*BPP. A) Schematic of the use of A*f*BPP in combination with an LC‒MS/MS approach to identify the targets of AA. B) Further evaluation of the labeling efficiency of mouse kidney proteins by AA‐P according to in‐gel fluorescence. C) The ratio plot for all AA‐interacting proteins identified with AA‐P. D) Venn diagrams showing the number of AA‐interacting proteins identified specifically with AA‐P. E) Bioinformatic analysis of AA‐interacting proteins. F) List of high‐confidence candidate target proteins of AA.

We next used the AA probe in a chemical proteomics approach to identify the direct targets of AA (**Figure** **4**A). We further confirmed that 50 µm AA‐P can label kidney proteins and that this labeling can be effectively blocked by incubation with 500 µm AA (Figure 4B). Next, kidney proteins were incubated with DMSO, AA‐P (50 µm), or AA‐P (50 µm) + AA (500 µm). Photo‐crosslinking and conjugation to biotin‐PEG3‐N_3_ were then performed, followed by incubation with neutravidin beads and stringent washing. The samples were digested to generate peptides that were analyzed via LC‒MS/MS (Figures S8–S25, Supporting Information). All the samples were analyzed in duplicate, and hits that were identified in three independent biological replicates were considered for further analysis. According to this criterion, when the AA‐P group was compared with the DMSO group, 114 target proteins were identified as direct binding targets of AA in mouse kidneys (Figure 4C). Among these proteins, 32 target proteins could be labeled by AA‐P, and this labeling could be blocked by AA (Figure 4C–E). These 32 proteins were classified into mitochondrion, lipase inhibitor activity, lysosome, retinoid metabolism and transport, and phase II‐conjugation of compounds. All these proteins are listed in Figure 4F, e.g., ATP5J2, MPC‐1, SLC25A3, COQ7, and HIGD1A.

### 2.5 HIGD1A Knockdown Exacerbated AA‐Induced Injury in HK‐2 Cells

**Figure 5:**
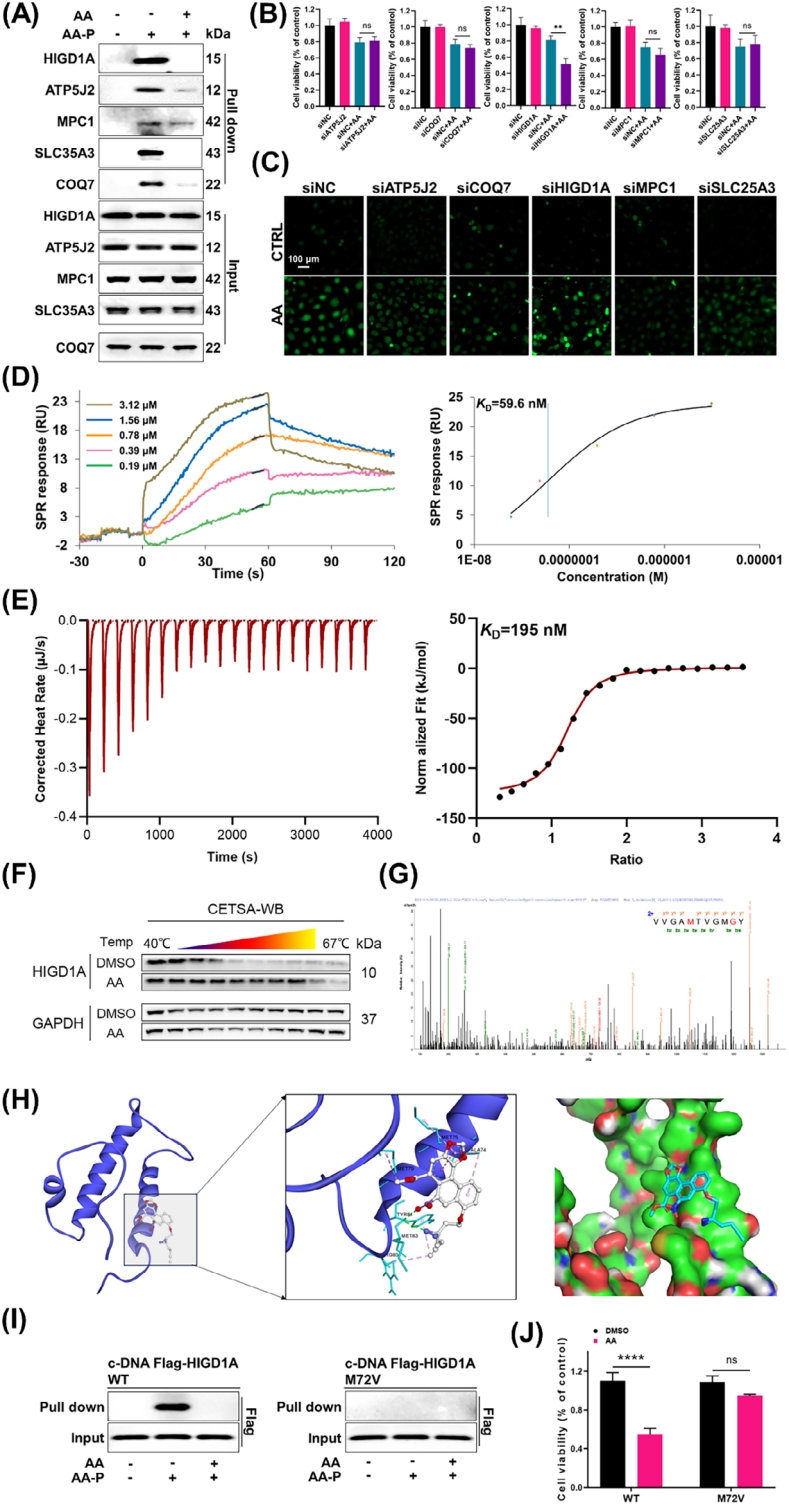
HIGD1A knockdown exacerbated AA‐induced mitochondrial injury in HK‐2 cells. A) Pull‐down analysis reveals the interaction between mitochondrial proteins and AA. B) Effects of ATP5J2, COQ7, HIGD1A, MPC1, or SLC25A3 knockdown on the viability of AA‐treated cells. C) Effects of ATP5J2, COQ7, HIGD1A, MPC1, or SLC25A3 knockdown on ROS production expression in HK‐2 cells. D,E) SPR and ITC analysis of AA binding to HIGD1A. F) The thermostability of HIGD1A was analyzed in HK‐2 cells that were treated or not with AA. G) LC‐MS/MS analysis of HIGD1A incubated with AA showed binding sites. H) Docking model of AA in the structure of HIGD1A. I,J) HIGD1A (M72V) is responsible for AA binding to HIGD1A. The effects of transfecting Flag‐tagged HIGD1A and its mutant plasmids on the viability of HK‐2 cells that were treated or not with AA. Student's *t*‐test is used to compare two groups of data affected by a single variable. All data are presented as mean ± standard deviation (*n* = 3). Differences were considered statistically significant at *p < 0.05, **p < 0.01, ***p < 0.001, ****p < 0.0001; ns was considered not significant.

Inspired by the A*f*BPP results, five proteins that are located in the mitochondria were selected, and their roles in AA‐induced injury were investigated. We first employed pull‐down analysis to assess the interaction between these proteins and AA. The pull‐down results showed that HIGD1A, ATP5J2, MPC1, SLC35A3, and COQ7 could be effectively pulled down in the presence of AA‐P (**Figure** **5**A). As shown in Figure S26 (Supporting Information), AA significantly altered the expression and distribution of the above‐mentioned proteins in murine renal tissues. Then we individually knocked down these five proteins in HK‐2 cells. We observed that depletion of HIGD1A, but not depletion of the other proteins, rendered the HK‐2 cells sensitive to AA treatment (Figure 5B). Consistent with these findings, HIGD1A knockdown further increased AA‐induced ROS production (Figure 5C). Combined with previous pull‐down assays and the knockdown results in this study, further establishes HIGD1A as a critical target protein in AA‐induced damage. To confirm the biophysical basis of the HIGD1A‐AA interaction, surface plasmon resonance (SPR) and isothermal titration calorimetry (ITC) were employed. Both SPR and ITC confirmed a direct binding event, revealing high‐affinity interactions with dissociation constants *K*_D_ of 59.6 nm (SPR) and 195 nm (ITC) (Figure 5D,E). Small molecules may increase the thermostability of proteins via the formation of a ligand‒protein complex.^[^ ^23^ ^]^ A CETSA‐WB experiment was performed to evaluate the effect of AA on HIGD1A stability. Compared with DMSO, AA significantly increased HIGD1A thermostability over a range of temperatures (40–67 °C) (Figure 5F). We subsequently employed LC‐MS/MS to identify the binding sites of AA‐P on HIGD1A. As shown in Figure 5G, peptide segments Val71‐Tyr80 of HIGD1A were identified as AA‐P‐binding regions (Figure 5G). Molecular docking simulations also elucidated the binding mode of AA to HIGD1A, revealing key interactions between AA and residues Met72 and Ala74 at the binding interface (Figure 5H). Figure S27 (Supporting Information) details the stabilizing intermolecular forces‐including hydrogen bonds and hydrophobic interactions‐involving residues Ala74, Met75, and Met83. To identify the critical binding sites of HIGD1A, Met72, Met75, and Met83 were individually mutated to valine. Pull‐down assays revealed that the M72 mutation markedly reduced the binding affinity of AA for HIGD1A (Figure 5I). To further validate the importance of Met72, we transfected HK‐2 cells with plasmids expressing either wild‐type HIGD1A or the HIGD1A (M72V) mutant. Notably, reintroduction of HIGD1A (M72V) significantly attenuated AA‐induced toxicity (Figure 5J). These findings demonstrate that AA binds directly to HIGD1A via Met72. Together, our results identify HIGD1A as a direct molecular target of AA and underscore its critical role in mediating AA‐induced kidney injury.

### 2.6 The Interaction Between the TFAM and HIGD1A was Inhibited by AA Treatment

**Figure 6:**
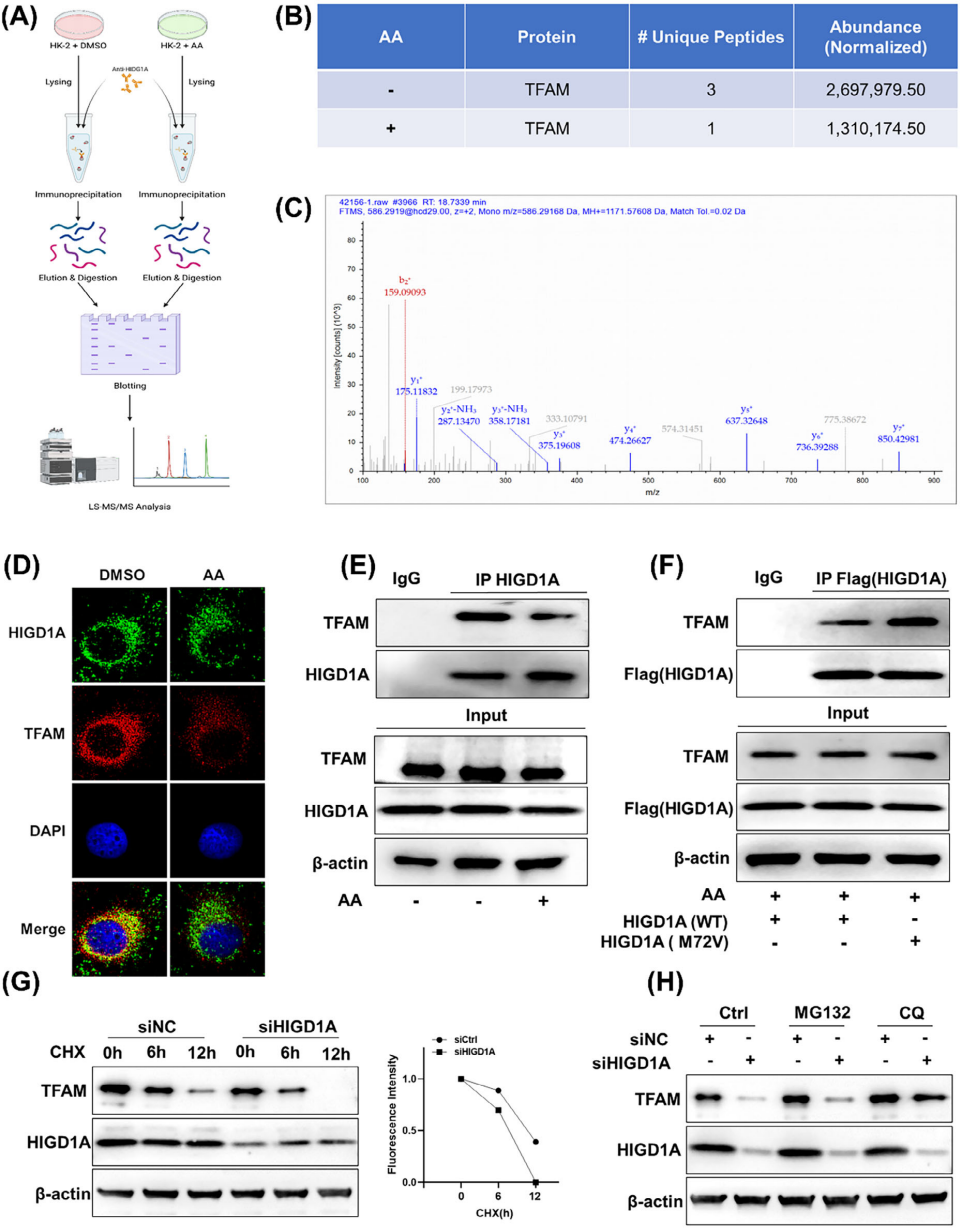
The interaction between the TFAM and HIGD1A was inhibited by AA treatment. A) Schematic illustration of quantitative proteomic screen to identify proteins binding to HIGD1A. B) LC‐MS/MS analysis of HIGD1A‐bound proteins from HK‐2 cell lysate. C) LC‐MS/MS analysis showed that HIGD1A binds to TFAM. HIGD1A was incubated with AA at 4 °C overnight. D) Co‐localization of HIGD1A (green) and TFAM (red) by immunofluorescence analysis. E) Immunoprecipitation of HIGD1A or control IgG was performed on HK‐2 cells treated with PBS or AA (100 µm) for 24 h. F) Met72 mutation restores the interaction of HIGD1A with TFAM. G) Effect of knockdown of HIGD1A on TFAM degradation. H) HK‐2 cells were treated with either MG132 (10 µm) or CQ (50 µm) before being harvested and were subsequently subjected to western blot analysis. All data are presented as mean ± standard deviation (*n* = 3).

**Figure 7:**
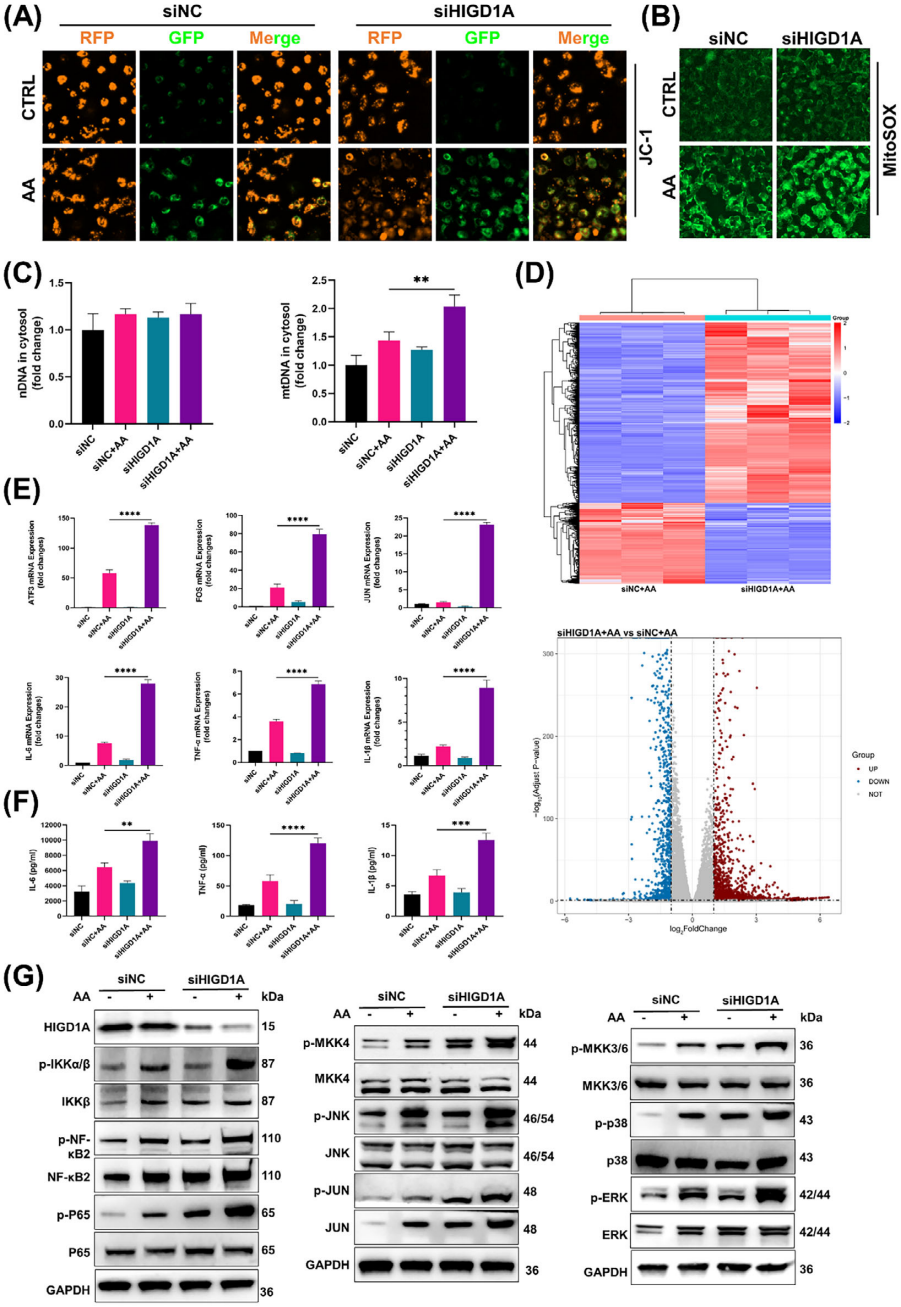
HIGD1A regulated AA‐induced mitochondrial dysfunction and inflammation in HK‐2 cells. A,B) Effects of AA on mitochondrial depolarization and mitochondrial superoxide production in HIGD1A‐ or NC‐knockdown HK‐2 cells. C) The levels of nuclear and mtDNA in the cytosol were determined. D) RNA‐seq results revealed that HIGD1A regulated the mRNA expression patterns in AA‐treated HK‐2 cells. E) RT‒qPCR confirmed the changes in the mRNA expression of ATF3, FOS, JUN, IL‐6, TNF‐α, and IL‐1β. F) The concentrations of IL‐6, TNF‐α, and IL‐1β were measured by ELISA. G) Western blotting analysis of MAPK and NF‐κB pathway component expression in siHIGD1A‐ or siNC‐transfected HK‐2 cells treated with or without AA. Student's *t*‐test is used to compare two groups of data affected by a single variable. All data are presented as mean ± standard deviation (*n* = 3). Differences were considered statistically significant at *p < 0.05, **p < 0.01, ***p < 0.001, and ****p < 0.0001.

To delineate the role of HIGD1A in AA‐induced mitochondrial injury, we performed co‐immunoprecipitation (Co‐IP) coupled with LC‐MS/MS to identify HIGD1A‐interacting proteins in HK‐2 cells. Mass spectrometry analysis revealed TFAM as a consistent HIGD1A interactor under both basal and AA‐exposed conditions (Figures**6**C; S28, Supporting Information). This direct interaction was further supported by immunofluorescence assays, which revealed significant colocalization of HIGD1A and TFAM (Figures 6D; S29, Supporting Information). Quantitative proteomics showed a 2.06‐fold higher TFAM abundance in Co‐IP complexes from untreated cells versus AA‐treated cells, suggesting impaired TFAM‐HIGD1A binding upon AA exposure (Figure 6B). We validated this disruption using Co‐IP assays, which confirmed that AA markedly inhibits the formation of the HIGD1A‐TFAM complex (Figure 6E). Based on the finding that AA binds to the Met72 residue of HIGD1A, we introduced an M72V point mutation to abrogate this interaction. Co‐IP analysis demonstrated that, in contrast to the wild‐type protein, the HIGD1A‐M72V mutant maintained a stronger interaction with TFAM even after AA treatment. This was supported by higher TFAM levels in the immunoprecipitates (Figure 6F), indicating that the Met72 site is essential for AA‐mediated dissociation of the HIGD1A‐TFAM complex. While HIGD1A knockdown via siRNA exacerbated AA‐mediated TFAM downregulation (Figures S30–S31, Supporting Information). Cycloheximide (CHX) chase assays demonstrated that AA reduced TFAM protein half‐life from 15.8 ± 0.3 h to 6.9 ± 0.4 h (**Figure** **7**G). Attenuation of TFAM degradation by chloroquine (CQ), an autophagy‐lysosome inhibitor, implicated autophagic flux as the primary TFAM degradation route (Figure 7H). These results demonstrated that AA dissociates the HIGD1A‐TFAM complex, leading to TFAM destabilization and accelerated autophagic degradation.

### 2.7 HIGD1A Regulated AA‐Induced Mitochondrial Dysfunction and Inflammation in HK‐2 Cells

**Figure 8:**
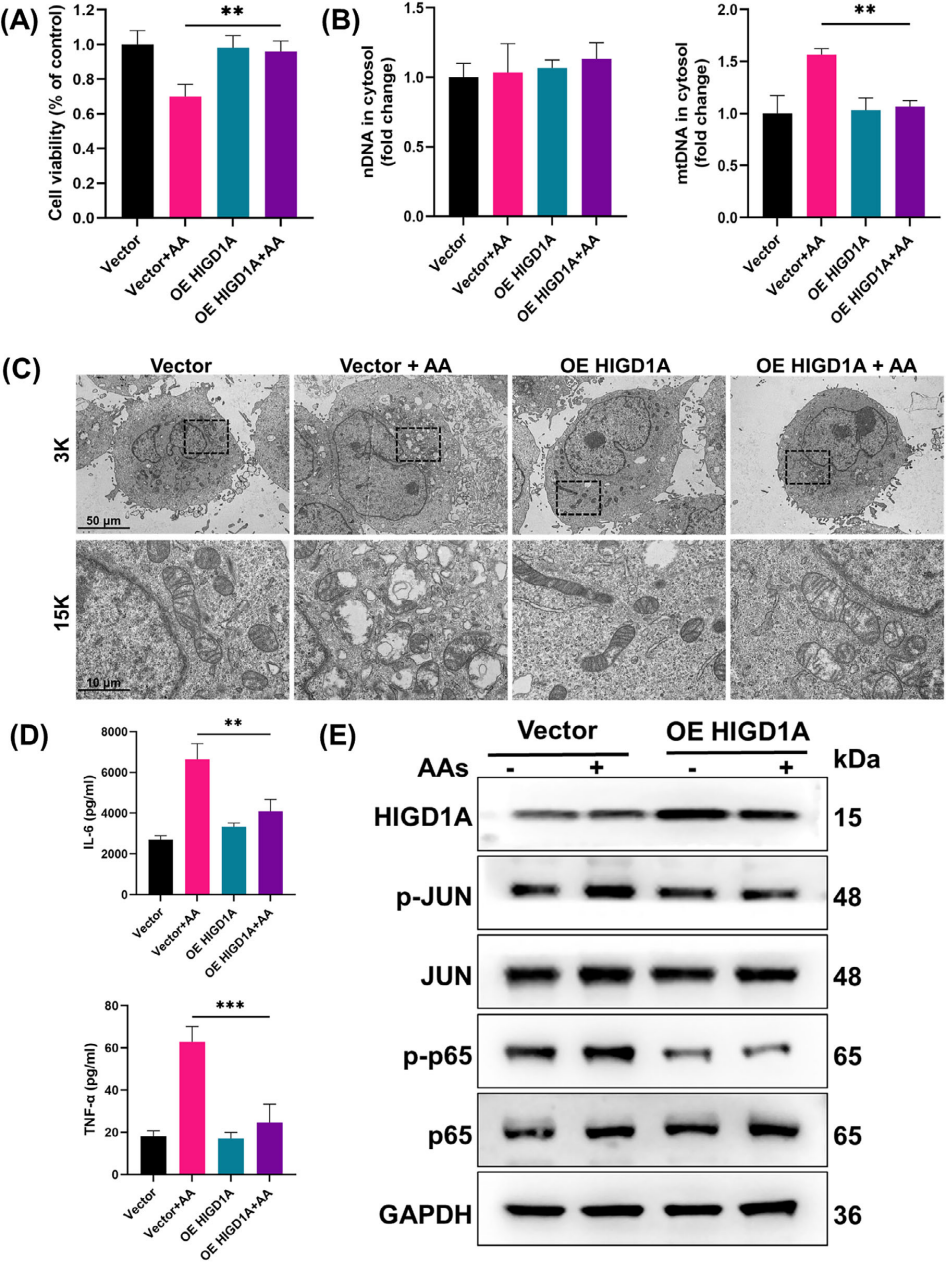
HIGD1A overexpression ameliorates AA‐induced injury in HK‐2 cells. A) Effects of HIGD1A overexpression on the viability of AA‐treated cells. B) The levels of nuclear and mtDNA in the cytosol were determined. C) Changes in the morphology and structure of mitochondria in OE HIGD1A or Vector HK‐2 cells were observed via transmission electron microscopy after AA treatment. D) The concentrations of IL‐6 and TNF‐α were measured by ELISA. E) Western blotting analysis of JUN and p65 expression in OE HIGD1A or Vector HK‐2 cells treated with or without AA. Student's *t*‐test is used to compare two groups of data affected by a single variable. All data are presented as mean ± standard deviation (*n* = 3). Differences were considered statistically significant at *p < 0.05, **p < 0.01, and ***p < 0.001.

Next, we aimed to explore how HIGD1A contributes to the toxic effects of AA. Since HIGD1A is a mitochondrial protein, we first explored the status of mitochondria by JC‐1 and MitoSox Green staining. As shown in Figures 7A,B and S32 (Supporting Information), HIGD1A knockdown significantly exacerbated the AA‐induced decrease in the MMP and further increased the AA‐induced mitochondrial superoxide production. The cytosolic levels of nuclear DNA (nDNA) and mtDNA were also measured. HIGD1A knockdown further increased the cytosolic level of mtDNA (Figure 7C). To further explore the role of HIGD1A in AA‐induced injury, RNA‐seq was performed to observe the differences in mRNA expression between the siNC+ AA group and the siHIGD1A + AA group. As shown in Figure 7D, after HIGD1A was knocked down, the levels of mRNAs, such as the JUN, FOS, ATF3, IL‐6, TNF‐α, and IL‐1β mRNAs, were significantly increased. We then confirmed the changes in the levels of these mRNAs (Figure 7E), and the results suggested that HIGD1A may also regulate AA‐induced inflammation via mtDNA release. We further measured the expression of IL‐6, TNF‐α, and IL‐1β in HK‐2 cells with ELISA kits. Compared with the AA group, HIGD1A knockdown further increased IL‐6, TNF‐α, and IL‐1β expression. Inspired by the results of RNA‐seq and RT‒qPCR, the expression levels of proteins in the MAPK, NF‐κB, and mTOR pathways were measured. Western blotting analysis of AA‐treated cells revealed that AA induced the phosphorylation of IKKα/β, NF‐κB2, and p65 (Figure 7G). Similar to NF‐κB signaling, HIGD1A knockdown further increased MAPK signaling pathway activation in AA‐treated cells. As shown in Figure 7G, AA treatment also promoted the phosphorylation of MKK4‐JNK, MKK3/6‐p38, and JUN. To further confirm the role of HIGD1A in AA‐induced toxicity, a HIGD1A plasmid was transiently transfected into HK‐2 cells to overexpress the HIGD1A protein. After 48 h, the cells were treated with AA (100 µM) and further incubated for 24 h. As shown in **Figure** **8**A, the HIGD1A‐overexpressing HK‐2 cells exhibited strong resistance to AA. The cytosolic level of mtDNA in the OE HIGD1A+AA group was significantly lower than that in the Vector +AA group (Figure 8B). The results of electron microscopy also suggested that AA treatment induced severe mitochondrial damage in Vector cells, characterized by swelling, vacuolization, cristae loss, and membrane rupture. Overexpression of HIGD1A significantly rescued these defects, maintaining mitochondrial integrity with preserved cristae architecture and minimal swelling in AA‐treated conditions (Figure 8C). Moreover, HIGD1A overexpression reversed the changes in IL‐6, TNF‐α, and IL‐1β production compared with those in the Vector +AA group (Figures 8D; S33, Supporting Information). We also measured the phosphorylation of JNK, p38, and JUN. HIGD1A overexpression reversed the AA‐induced phosphorylation of JNK, p38, and JUN (Figure 8E).

## 3 Discussion

In terms of traditional Chinese medicine, acute kidney injury, toxicology, and tumorigenesis, few compounds have attracted as much attention as aristolochic acids.^[^ ^24^ ^]^ Most studies have focused on aristolochic acid‐induced DNA damage, and few researchers have explored the direct targets of aristolochic acid.^[^ ^9^, ^25^, ^26^ ^]^ In this study, we designed and synthesized an AA‐based affinity probe, AA‐P, that exerted toxic effects that were similar to those of aristolochic acids. Using AA‐P, we identified 32 AA‐interacting proteins, including ATP5J2, COQ7, HIGD1A, and Bax. Among these proteins, HIGD1A demonstrated a high affinity for AA. We further demonstrated that HIGD1A regulates AA‐induced mitochondrial dysfunction and inflammation, with a novel mechanism involving the disruption of the HIGD1A‐TFAM complex as a central driver of toxicity.

It is important to explore the direct target proteins of small‐molecule toxic chemicals. According to the screening techniques that are used, most toxicological studies can be classified into two different types. 1) The target proteins of toxic chemicals are selected from among classic proteins, such as NRF2, NLRP3, PARP, and eNOS.^[^ ^27^, ^28^, ^29^, ^30^, ^31^ ^]^ These classic proteins are generally sensitive to external stimuli, e.g., toxic chemicals, and the knockdown or overexpression of these proteins often alleviates the damage that is caused by toxic chemicals.^[^ ^27^, ^28^, ^30^, ^31^ ^]^ The functions and expression of these proteins have been verified for nearly all important toxic chemicals.^[^ ^12^, ^32^, ^33^ ^]^ The disadvantage of this method is the lack of systems and the difficulty in identifying the real targets of toxic chemicals. 2) Omics is a general technique that is currently used for toxicological studies.^[^ ^34^, ^35^, ^36^, ^37^ ^]^ Various mRNAs, proteins, and metabolites that are targeted by toxic chemicals can be identified via omics.^[^ ^34^, ^35^, ^36^ ^]^ However, the amount of work that is needed to verify omics results is substantial. Furthermore, most toxic chemicals directly affect protein activity via direct interaction with proteins and do not alter protein expression in a short time; this effect of toxic chemicals cannot be detected by omics.^[^ ^38^, ^39^, ^40^ ^]^ Because there are few other methods for screening target proteins of toxic chemicals, more suitable methods are urgently needed for the toxicological study of small‐molecule toxic chemicals. As mentioned in the introduction, A*f*BPP is a classic technique for identifying target proteins of chemical drugs and endogenous biological molecules in drug development and chemical biology.^[^ ^21^, ^22^ ^]^ We propose that this technique could also be adapted to systematically identify direct cellular targets of small‐molecule toxic chemicals.

On the basis of the mapping of AA‐interacting proteins, we further identified HIGD1A, which is closely related to AA‐induced toxicity. HIGD1A is a mitochondrial inner membrane protein with a molecular weight of ≈10.4 kDa.^[^ ^41^ ^]^ It has been reported that HIGD1A regulates ROS production by affecting the MMP.^[^ ^42^ ^]^ In our study, HIGD1A knockdown decreased the MMP and increased ROS production in the context of AA exposure, whereas HIGD1A overexpression inhibited AA‐induced mitochondrial dysfunction; this finding is consistent with a previous report. The SPR results also revealed the high affinity between HIGD1A and AA (SPR: 59.6 nm; ITC: 195.0 nm), as did the results of the CETSA‐WB experiments. Furthermore, our study also uncovers a novel pathogenic mechanism: AA binding to HIGD1A disrupts the HIGD1A‐TFAM complex, triggering TFAM destabilization and subsequent autophagic‐lysosomal degradation. This may lead to the accumulation of cytosolic mtDNA. We propose that HIGD1A is an important protein target of AA and plays a vital role in AA‐induced mitochondrial dysfunction.

Inflammation also plays an important role in AA‐induced nephropathy.^[^ ^14^, ^43^ ^]^ We observed that HIGD1A knockdown could cause a more than ten‐fold increase in the expression of some inflammation‐related mRNAs, e.g., ATF3, FOS, JUN, and IL‐6, under conditions of AA exposure, as determined by RNA‐seq and RT‐qPCR. Additionally, some groups have reported that AA can increase the expression of p‐ERK, p‐JNK, and p‐p38 and that these changes are caused mainly by the AA‐induced damage to nuclear DNA.^[^ ^18^, ^20^ ^]^ Our results also confirmed the increase in inflammatory indicators, such as p‐ERK, p‐JNK, and P‐p38, after AA exposure. Furthermore, we identified an additional mechanism centered on the binding of AA to HIGD1A. While nuclear DNA damage contributes to inflammation, our findings suggest that the increase of these inflammation‐related indicators is also linked to the binding of AA to HIGD1A. We found that AA could increase the cytosolic level of ox‐mtDNA and that HIGD1A overexpression could reverse the change in the cytosolic level of ox‐mtDNA. While HIGD1A knockdown can increase the cytosolic level of mtDNA in AA‐treated HK‐2 cells, which is closely related to inflammation.^[^ ^44^ ^]^ The Zhao group also reported significant synergistic effects of HIGD1A and DNA damage in other models.^[^ ^45^ ^]^ We propose that the binding of AA to HIGD1A and nuclear DNA damage jointly amplify inflammatory cascades in AA nephropathy. This may also partly explain why AA exhibits nanomolar affinity for HIGD1A, yet requires micromolar concentrations to elicit functional changes in HK‐2 cells. Although nanomolar AA binds HIGD1A and alters its function, micromolar concentrations are necessary to induce DNA damage.

There are several limitations to this study. First, this study focused mainly on AA‐interacting mitochondrial proteins, and several important proteins, e.g., NGAL, Bax, and APOA1, should be investigated in future studies. Second, the exact AA‐binding residues within HIGD1A's Val71‐Tyr80 peptide segment necessitate validation. Third, while the functional role of HIGD1A was validated in HK‐2 and HEK293T cells, the mechanistic studies exploring HIGD1A in AA toxicity were primarily conducted in HK‐2 cells; these findings should be validated across multiple renal cell types to ensure broader relevance. Finally, HIGD1A‐knockdown and HIGD1A‐overexpressing cells were established to explore the mechanism of AA action, but the lack of knockdown/overexpressing mice prevented further verification of the functional role of HIGD1A in the mechanism underlying AA toxicity.

## 4 Conclusion

In summary, we developed a clickable, photoreactive AA affinity‐based probe AA‐P, and evaluated its toxicity in living cells and mice. Employing this probe, we systematically mapped AA‐interacting proteins in murine kidneys, identifying HIGD1A as a high‐affinity target critically involved in AA‐induced mitochondrial injury. Furthermore, we demonstrated that AA binding to HIGD1A specifically disrupts the HIGD1A‐TFAM complex, triggering TFAM degradation via autophagic‐lysosomal pathways. The ensuing destabilization of TFAM underpinned AA‐induced mitochondrial dysfunction and drives inflammation through hyperactivation of MAPK/NF‐κB pathways. This study not only provided the comprehensive AA‐interactome atlas but also identified HIGD1A‐TFAM complex stabilization as a novel therapeutic target against AA nephrotoxicity.

## 5 Experimental Section

The Committee on Ethics of Medicine, Naval Medical University, PLA approved this study, and a certificate of approval is available upon request. Animal experiments were performed according to the regulations and guidelines approved by the Animals Research Committee at Naval Military Medical University (SMMU, Licence No. 2011023, NMUMREC‐2021‐013). The animals received humane care throughout the procedures in accordance with the Guide for the Care and Use of Laboratory Animals published by the US National Institutes of Health (publication no. 85‐23, revised 1996).

### Materials

All these experimental materials, including Aristolochic acid (Macklin, #A800933), 5‐TAMRA‐azide (Confluore, #BDR‐2), Biotin‐PEG3‐azide (Confluore, #BCA‐21), BTTAA (Confluore, #BDJ‐4), Ammonia solution (SupeIco, #Z0726130118), Detergent Compatible Bradford Protein Assay Kit (Beyotime, #P0006C), Calcium chloride dihydrate (InnoChem, #A73062), 3‐[4‐(2‐Hydroxyethyl)piperazin‐1‐yl] propanesulfonic acid (Sigma, #E9502), cOmplete, EDTA‐free Protease Inhibitor (Sigma–Aldrich, #4693132001), Detergent Compatible Bradford Protein Assay Kit (Beyotime, #P0006C), DL‐Dithiothreitol (InnoChem, #A32496), Dulbecco's Phosphate Buffered Saline (Thermo Fisher, #14190250), Ethanol (InnoChem, #KDGED07), HEPES (Sigma–Aldrich, #V900477‐100G), Hydroxylamine solution (Aldrich, #STBJ9216), Iodoacetamide (Sigma–Aldrich, #I1149), Lysyl Endopeptidase®Mass Spectrometry Grade (Wako, #125‐05061), Sequencing Grade Modified Trypsin (Promega, #V5111), Sodium chloride (Sigma–Aldrich, #S7653‐250G), Sodium dodecyl sulfate (Sigma–Aldrich, #71725), Dynabeads^TM^ M‐280 Streptavidin (ThermoFisher, #LT‐02241), TMT sixplex^TM^ Label Reagent Set (Thermo,#90061), Triethylammonium bicarbonate buffer (Sigma–Aldrich, #18597), Urea (Sigma–Aldrich, #U5378), IL‐6 assay kit (Westang, #F01310), IL‐8 assay kit (Westang, #F01340), TNF‐α assay kit (Westang, #F02810), IL‐1β assay kit (Westang, #F01220), Pierce Pull‐Down Biotinylated Protein:Protein Interaction Kit (Thermo Fisher, #21115), Alexa Fluor™ 488 (Thermo Fisher, #A‐11008), Alexa Fluor™ 647 (Thermo Fisher, #A‐31571), Anti‐p‐IKKα/β (Cell Signaling Technology, #2697P), Anti‐IKKβ (Cell Signaling Technology, #2678), Anti‐p‐NF‐κB2 (Cell Signaling Technology, #4810P), Anti‐NF‐κB2 (Cell Signaling Technology, #3017), Anti‐p‐P65 (Cell Signaling Technology, #3033), Anti‐P65 (Cell Signaling Technology, #4764), Anti‐GAPDH (Abclonal Technology, #AC010), Anti‐HIGD1A (Proteintech, #21749‐1), Anti‐p‐MKK4 (Cell Signaling Technology, #9156L), Anti‐MKK4 (Cell Signaling Technology, #9152), Anti‐p‐JNK (Cell Signaling Technology, #9251L), Anti‐JNK (Cell Signaling Technology, #9252), Anti‐p‐MKK3/6 (Cell Signaling Technology, #9231L), Anti‐MKK3 (Cell Signaling Technology, #9232), Anti‐p‐JUN (Cell Signaling Technology, #3270), Anti‐JUN (Cell Signaling Technology, #9165), Anti‐p‐P38 (Cell Signaling Technology, #4631S), Anti‐P38 (Cell Signaling Technology, #9212), Anti‐p‐mTOR (Cell Signaling Technology, #2971), Anti‐mTOR (Cell Signaling Technology, #2972), Anti‐P‐4E‐BP1 (Cell Signaling Technology, #9451), Anti‐4E‐BP1 (Cell Signaling Technology, #9644), were purchased from commercial sources and used without further purification or modification unless otherwise stated.

### Cell Culture

HK‐2 human proximal tubular epithelial cells (RRID: CVCL_0302) and HEK293T human embryonic kidney cells (RRID: CVCL_0063) were purchased from the Shanghai Institute of Biochemistry and Cell Biology, Chinese Academy of Sciences. Short tandem repeat (STR) profiling and Mycoplasma testing was performed by Procell Life Science & Technology Co., Ltd., within 6 months (please refer to supporting information‐Reports of STR profiling and Mycoplasma testing). Cells were thawed and kept at low passages (≤20) to prevent genetic drift. These cells were cultured in low‐glucose DMEM supplemented with 10% fetal bovine serum, 100 U mL^−1^ penicillin, and 100 U mL^−1^ streptomycin and maintained in an incubator with 5% CO_2_ at 37 °C. The cells were regularly passaged and grown to 80–90% confluence before treatment.

### Detection of Cell Viability

HK‐2 cells were seeded in 96‐well plates at a density of 1 × 10^4^ cells per well and incubated for 24 h. Then, the cells were treated with different concentrations of AA or AA‐P for 24 h. Cell viability was assayed using a CCK‐8 Kit according to the manufacturer's instructions by measuring absorbance at 450 nm. The cell survival rate in each group was calculated according to the following formula: cell viability = (experimental group absorbance ‐ blank group) / (control group absorbance ‐ blank group) × 100%.

### Animal Experiments

The animals that were used in the experiment were SPF‐grade healthy female C57BL/6 mice (6–8 weeks old) that were provided by the Laboratory Animal Center of Naval Medical University. The animal protocol was approved by the Animal Protection and Use Committee of Naval Medical University, and the animal experiments were conducted in accordance with the provisions of Chinese laws on the use of laboratory animals. All the mice were maintained in a clean environment with free access to food, a normal circadian rhythm, a temperature of 22 ± 2 °C, and a humidity of 50% ± 10%; additionally, the mice were protected from strong light and noise, and they were fed for at least 7 days before the experiment to allow them to adapt to the environment. The animals were used in accordance with animal ethical guidelines. For AA toxicity evaluation, a total of 30 mice were randomly divided into 2 groups, with 15 mice per group. The mice were injected i.p. with 20 mg kg^−1^ b.w. 0.9% N.S. or AA on day 0 and then randomly sacrificed on days 3, 5, and 7. Blood, kidney, and liver samples were collected. The blood was incubated at 4 °C overnight and centrifuged at 4 °C at 3000 rpm for 15 min, after which the supernatant was collected for biochemical investigations. A portion of the liver and one side of the kidney were fixed in 4% paraformaldehyde to generate paraffin sections, which were subjected to immunohistochemistry, and the remaining samples were frozen at −80 °C. For AA‐P toxicity evaluation, a total of 15 mice were randomly divided into 2 groups, with 5 mice per group. The mice were injected i.p. with 20 mg kg^−1^ b.w. 0.9% N.S., AA or AA‐P on day 0 and then sacrificed on day 7. Blood and kidney samples were collected. The blood was incubated at 4 °C overnight and centrifuged at 4 °C at 3000 rpm for 15 min, after which the supernatant was collected for biochemical investigations.

### Synthesis of AA‐P

To a solution of 1 (250 mg, 0.764 mmol) in DCM (6 mL) was added SOCl_2_ (136 mg, 1.146 mmol) dropwise at 0 °C. The reaction mixture was stirred at room temperature for 1 h. After completion of the reaction, triethylamine (154 mg, 1.528 mmol) in MeOH (4 mL) was added to the reaction mixture at 0 °C. Then, the mixture was concentrated. The residue was purified by column chromatography on silica gel (petroleum ether/EtOAc = 50/1) to yield an intermediate product (120 mg, 46%). The intermediate product (120 mg, 0.35 mmol) and K_2_CO^3^ (120.75 mg, 0.875 mmol) were suspended in DMF (2.5 mL). Then diazirine‐iodo (112.87 mg, 0.455 mmol) was added, and the reaction mixture was stirred for 3 h at 40 °C. After cooling to room temperature, 50 mL of water was added to the reaction mixture. Then, the mixture was extracted with 30 mL of EtOAc three times, and the layers were separated. The organic layer was subsequently washed with aqueous saturated brine solution and dried over Na_2_SO_4_. The organic layer was concentrated under reduced pressure. The crude material was purified by column chromatography on silica gel (petroleum ether/EtOAc = 10/1) to yield the product ACC‐1, which was a yellow solid (65 mg, 40%). ^1^H NMR (400 MHz, CDCl_3_) δ 8.57–8.51 (m, 1H), 7.75–7.66 (m, 2H), 7.36 (s, 1H), 7.27–7.24 (m, 1H), 6.37 (s, 2H), 4.05 (t, J = 12 Hz, 2H), 3.99 (s, 3H), 2.18–2.10 (m, 2H), 2.05–1.95 (m, 3H), 1.82 (t, J = 16 Hz, 2H). ^13^C NMR (101 MHz, CDCl_3_) δ 170.76, 157.45, 145.25, 144.94, 128.46, 127.74, 126.28, 125.75, 125.61, 123.32, 118.21, 117.99, 110.65, 109.58, 102.17, 82.74, 69.25, 62.77, 52.89, 32.93, 32.72, 26.71, 13.35.

### Cellular Imaging of AA‐P

HK‐2 cells were seeded in 96‐well plates at a density of 1 × 10^4^ cells per well and incubated for 24 h. Then, the cells were incubated in the dark with different concentrations of AAI‐P for 2 h. Then, the cells were irradiated with ultraviolet light for 10 min on ice. After that, the cells were fixed with 4% paraformaldehyde at room temperature for 15 min. After fixation, 0.3% Triton X‐100 was added to permeabilize the cells. Each group was conjugated with a fluorescence reporter by click chemistry in the dark for 1 h (100 µm rhodamine‐azide, 100 µm TBTA, 1 mm CuSO_4_, and 1 mm TCEP). Finally, the cells were incubated with Hoechst for 10 min in the dark. All the images were acquired via fluorescence microscopy (Agilent BioTek Cytation 5 Cell Imaging Multimode Reader).

### Fluorescence Labeling Experiments in SDS‐PAGE Gels

The samples were thawed on ice, and lysis buffer was added, followed by ultrasonic disruption. The lysate that was obtained from ultrasonic lysis was subsequently centrifuged. The supernatant was collected, and the protein concentration was quantified and adjusted to 2 mg mL^−1^. Then, 0.5 µL of DMSO, AA‐P, or AA‐P+AA were added to 50 µL of the protein mixture, and the mixture was subsequently centrifuged. The combined solution was incubated at 25 °C for 1 h. The sample was then placed on ice and exposed to UV light for 15 min. After UV light exposure, TAMRA‐azide, BTTAA/CuSO_4_ premix, and ascorbate were added. The mixture was agitated at 25 °C for 1 h. Protein loading buffer was added, and the sample was incubated at 95 °C for 5 min and then cooled. A total of 40 µL of the sample was loaded onto a 10% SDS‒PAGE gel for electrophoresis. The fluorescent signal was detected via a ChemiDoc MP, and the protein was stained with Coomassie blue staining solution.

### Pull‐Down and LC‒MS/MS‐Based Target Identification

The samples were thawed on ice, and lysis buffer was added, followed by ultrasonic disruption. This portion of the study was performed with support from ChomiX Biotech Co., Ltd. The lysate that was obtained from ultrasonic lysis was subsequently centrifuged. The supernatant was collected, and the protein concentration was quantified and adjusted to 2 mg mL^−1^. Then, 0.5 µL of DMSO, AA‐P, or AA+AA‐P were added to 50 µL of the protein mixture, and the mixture was subsequently centrifuged. The combined solution was incubated at 25 °C for 1 h. The sample was then placed on ice and exposed to UV light for 15 min. The sample was then supplemented with biotin‐PEG_3_‐azide, BTTAA/CuSO_4_ premix, and ascorbate. The mixed solution was reacted with agitation at 25 °C for 1 h. The protein was then precipitated with methanol‐chloroform. The protein precipitate was resuspended in 750 µL of 0.2% SDS/PBS and diluted with PBS to 0.05% SDS/PBS. Two hundred microliters of beads were added to each sample, and enrichment was conducted at room temperature for 3 h. After enrichment, the sample was washed with 0.05% SDS/PBS for 10 min at room temperature, followed by three washes with PBS, each lasting 3 min. The mixture was then washed three times with 1 mL of 100 mm TEAB, each lasting 3 min. The supernatant was discarded. The sample was reduced with dithiothreitol (500 mm) and alkylated with iodoacetamide (500 mm). Trypsin (0.5 µg µL^−1^) was added to the sample to generate the peptide solution. The solution was analyzed by a Q Exactive HF‐X Orbitrap mass spectrometer (Thermo Fisher Scientific).

### Cellular Thermal Shift Assay

Soluble proteins were extracted from HK‐2 cells with RIPA lysis buffer. Equal amounts of protein were incubated with AA or DMSO at 37 °C for 1 h prior to the CETSA heat pulse and aliquoted into PCR tubes. The samples were subsequently incubated at different temperatures on a Thermal Cycler C1000 Touch (Bio‐Rad, USA). The samples were subsequently centrifuged, and the supernatants were added to 5× loading buffer and then denatured (100 °C for 15 min). Finally, the Western blots were analyzed via ImageJ software.

### Western Blotting

Total protein was extracted from HK‐2 cells with RIPA lysis buffer. Proteins were separated by instant SDS‒PAGE (GenScript, China) for 0.5 h at 180 volts (V). The samples were further electrotransferred to polyvinylidene fluoride (PVDF) membranes for 1 h at 300 mA. The membranes were subsequently incubated with the relevant primary antibodies (1:500–1000). After being washed with TBST three times for 10 min each, the membranes were incubated with the relevant secondary antibodies (1:50000) for 1 h at RT and then washed three times with TBST. The signals were visualized via enzyme‐linked chemiluminescence (ECL) (Thermo Fisher, USA). The protein bands were analyzed via ImageJ software.

### Quantitative Real‐Time PCR

Total RNA was extracted with TRIzol (Invitrogen, Carlsbad, CA, USA) and reverse‐transcribed into cDNA with a PrimeScript RT kit (Vaztme, China) according to the manufacturer's instructions. Fluorescence quantitative PCR was performed according to the manufacturer's instructions for the SYBR Premix Ex Taq II kit (RR820A, TaKaRa) on a real‐time fluorescence quantitative PCR instrument (ABI 7500, ABI, Foster City, CA, USA). GAPDH was used as an internal reference to calculate the relative expression of each target gene. Each experiment was repeated three times. The relevant primers were designed by Sangon (Shanghai, China). The sequences of the genes that were used are provided in Table S1 (Supporting Information).

### Molecular Docking

The crystal structures of the proteins were retrieved from the protein data bank (HIGD1A: 2LOM). AutoDock 4.2 was used for molecular docking, and PyMoL was used for mapping. The red dashed lines represent hydrogen bonds, whereas the gray dashed lines represent nonpolar interactions.

### RNA Interference and Transfection

HK‐2 cells were transfected with siNC, siATP5J2, siCOQ7, siHIGD1A, siMPC1, or siSLC25A3 (GenePharma, Shanghai, China). A Lipofectamine 2000 transfection kit (Thermo Fisher Scientific, USA) was utilized for transfection, and all the transfection procedures were carried out strictly according to the manufacturer's instructions. The transfected cells were cultured with serum‐free DMEM in a constant‐temperature incubator with 5% CO_2_ at 37 °C for 48 h. The sequences of the siRNAs that were used are provided in Table S2 (Supporting Information).

### Cell Transfection and Transient Expression

A total of 150 µL of Opti‐MEM with 9 µL of Lipofectamine 3000 was mixed and added to each six‐well plate. Then, 150 µL of Opti‐MEM and 3 µL of 10 µm HIGD1A/vector plasmid were added to the six‐well plate and incubated for 5 min. After further incubation for 30 min, the DNA–liposome complexes were added to the cells. After 6 h of incubation at 37 °C, 1 mL of maintenance medium was added, and the mixture was incubated for an additional 48 h. Each plasmid transfection was performed in triplicate. Expression levels were monitored via Western blotting.

### Statistical Analysis

All statistical analysis was performed using GraphPad Prism 7.0 software. The data shown in the study were obtained from at least three independent experiments and all data in different experimental groups were expressed as mean ± SD (standard deviation). One‐way ANOVA with Tukey's multiple comparison test was used for analyses. Details of each statistical analysis were provided in the figure legends. Differences with *p*‐values < 0.05 were considered statistically significant, all statistical methods, sample sizes (*n*), and exact *p*‐values were specified in the respective figure legends.

## Conflict of Interest

The authors declare no conflict of interest.

## Supporting information



Supporting Information

## Data Availability

The data that support the findings of this study are available from the corresponding author upon reasonable request.
