# Supplementary material for: Affinity‐Based Protein Profiling Revealed that HIGD1A is a Direct Target Protein of Aristolochic Acids
Source: Adv Sci (Weinh). 2025 Nov 5;13(5):e13117. doi: 10.1002/advs.202513117 (PMC12850329; doi:10.1002/advs.202513117)

**Affinity-based Protein Profiling Revealed that HIGD1A is a Direct Target Protein of Aristolochic Acids**

Yin Gong ^a, b,1^, Shanshan Zhang ^a,1^, Anying Wei ^a, b,1^, Xiang Ji ^c,1^, Xiaodan Chong ^e,1^, Jinfeng Cen ^a^, Xuan Zhao ^a^, Zimeng Luo ^a^, Zhipeng Pei ^a^, Guanchao Mao ^a^, Xinkang Zhang ^a^, Mingxue Sun ^a^, Zhiguo Sun ^d,^ *, Zifei Yin ^c,^ *, Zhengrong Zou ^b,^ *, Wen-Qi Meng ^a,^ *

^a^ Faculty of Naval Medicine, Naval Military Medical University, Shanghai, China. E-mail: wenqimeng@smmu.edu.cn

^b^ College of Life Sciences, Jiangxi Normal University, Nanchang, 330022, China Email: zouzhr@jxnu.edu.cn

^c^ School of Traditional Chinese Medicine, Naval Medical University, Shanghai 200433, China Email: yinzifei870730smmu@163.com

^d^ Department of Pharmaceutical Science, Faculty of Pharmacy, Naval Medical University E-mail: sunzg1978@163.com

^e^ Clinical Cancer Institute, Translational Medicine Center, Faculty of Pharmacy, Naval Medical University

^1^These authors contributed equally to this work.

*Corresponding author.


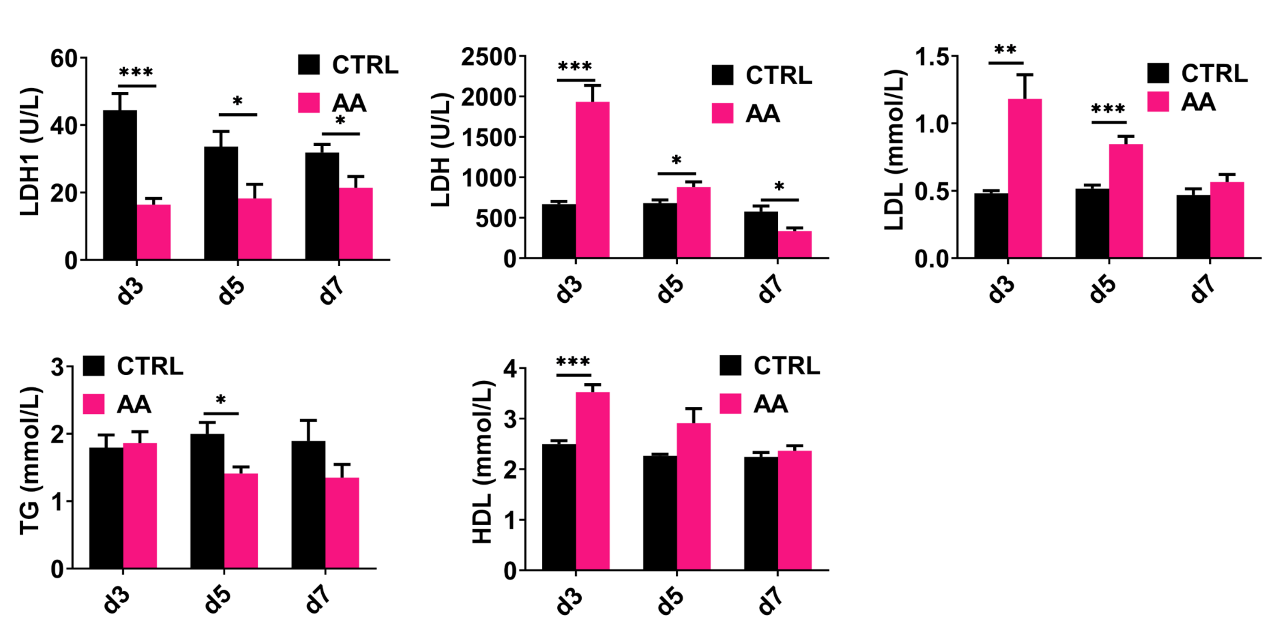


**Figure S1.** Effects of AA on the levels of serum indices of the mice. Student's t-test is used to compare two groups of data affected by a single variable. For the comparison of multiple groups of data, all data are presented as mean ± standard deviation (n = 3). Differences were considered statistically significant at **p* < 0.05, ***p* < 0.01, ****p* < 0.001, and *****p* < 0.0001.


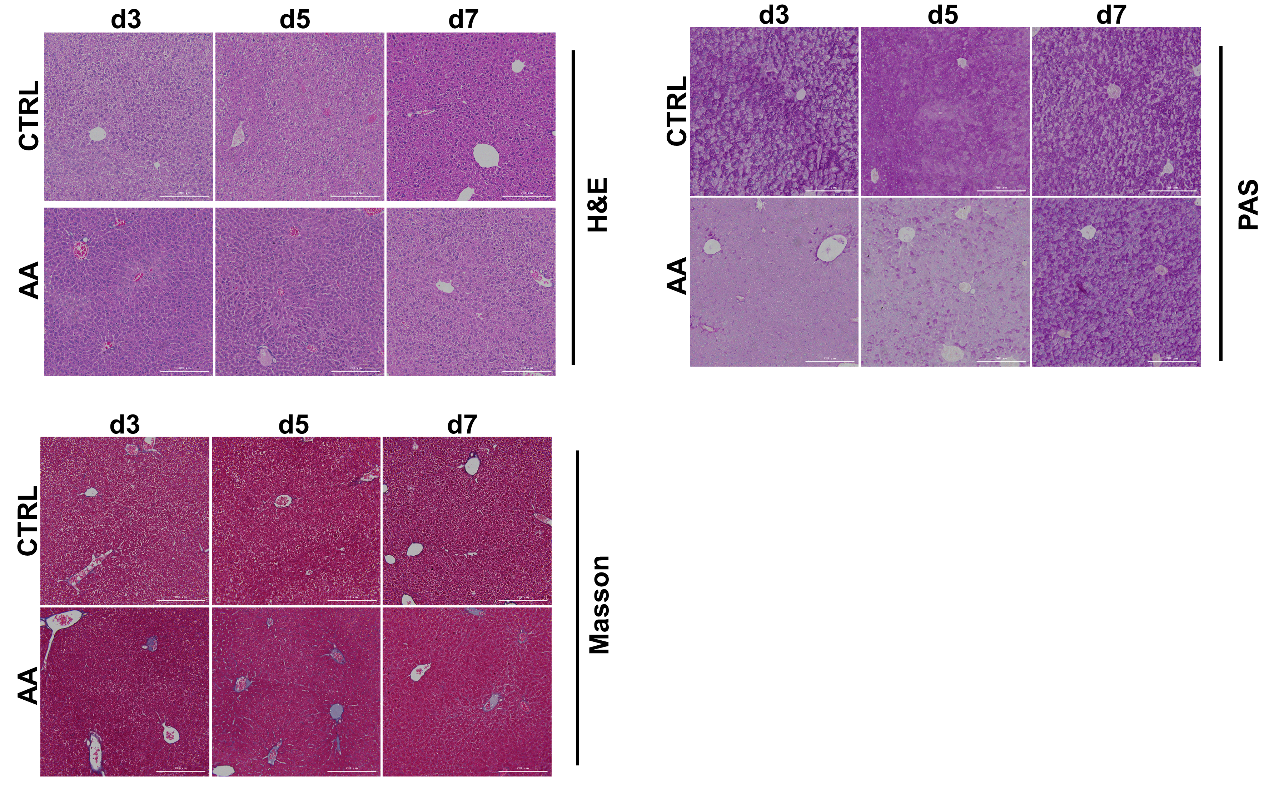


**Figure S2.** Mouse liver sections subjected to H&E, PAS and Masson’s trichrome staining after treatment with or without AA.


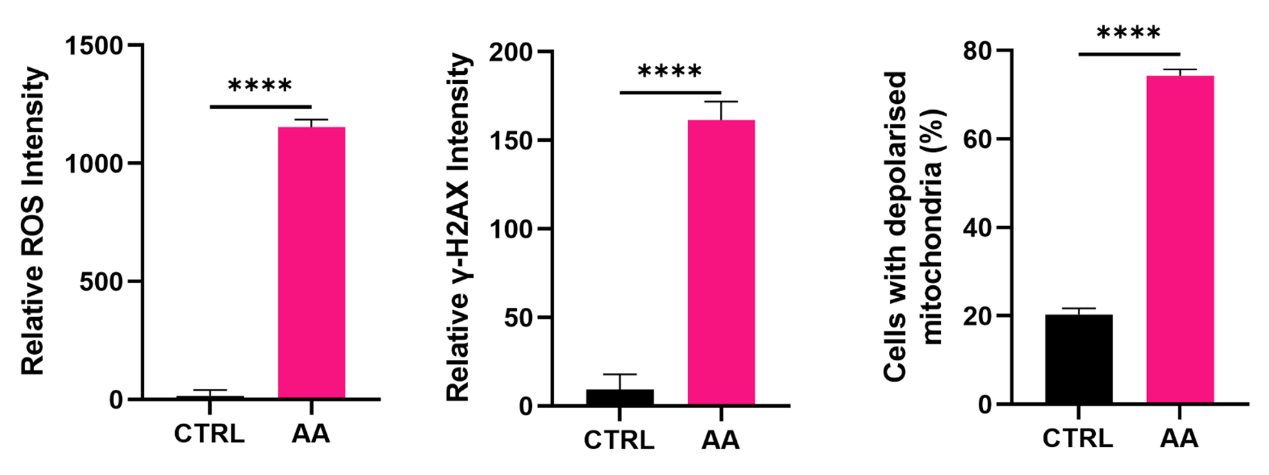


**Figure S3.** Quantification of the fluorescence intensities of ROS production, mitochondrial depolarization, and γ-H2A expression in HK-2 cells. Student's t-test is used to compare two groups of data affected by a single variable. All data are presented as mean ± standard deviation (n = 3). Differences were considered statistically significant at **p* < 0.05, ***p* < 0.01, ****p* < 0.001, and *****p* < 0.0001.


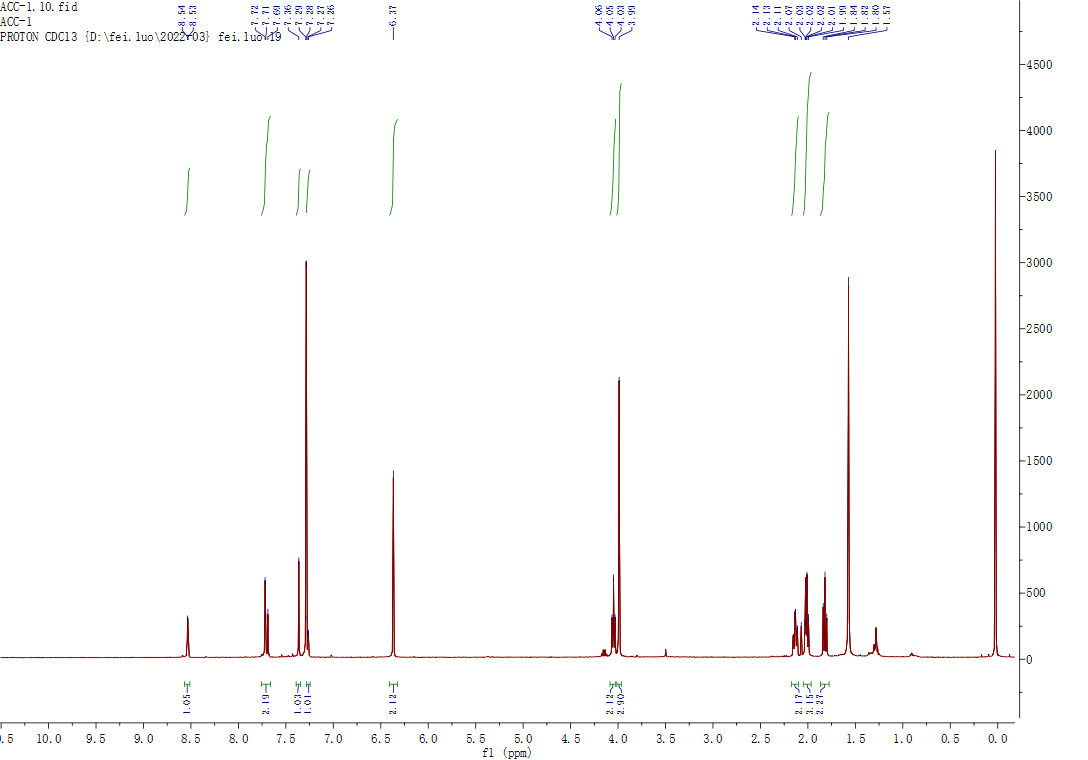


**Figure S4.** ^1^HNMR of AA-P in CDCl_3_.


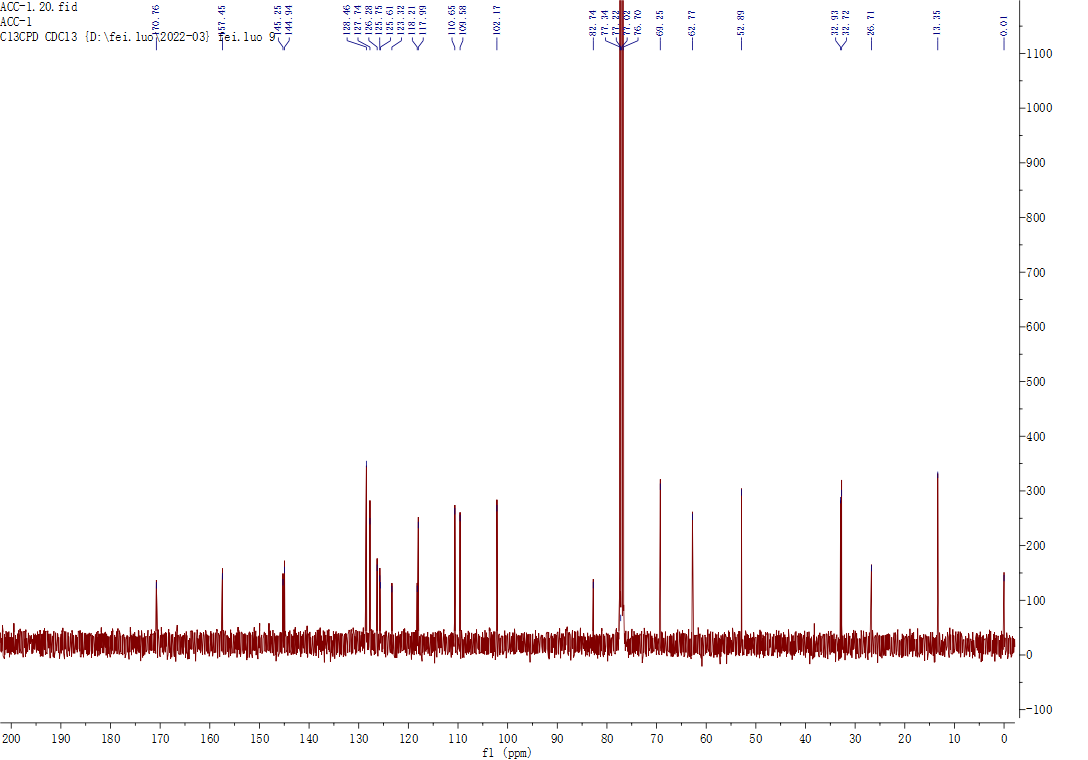


**Figure S5.** ^13^CNMR of AA-P in CDCl_3_**.**


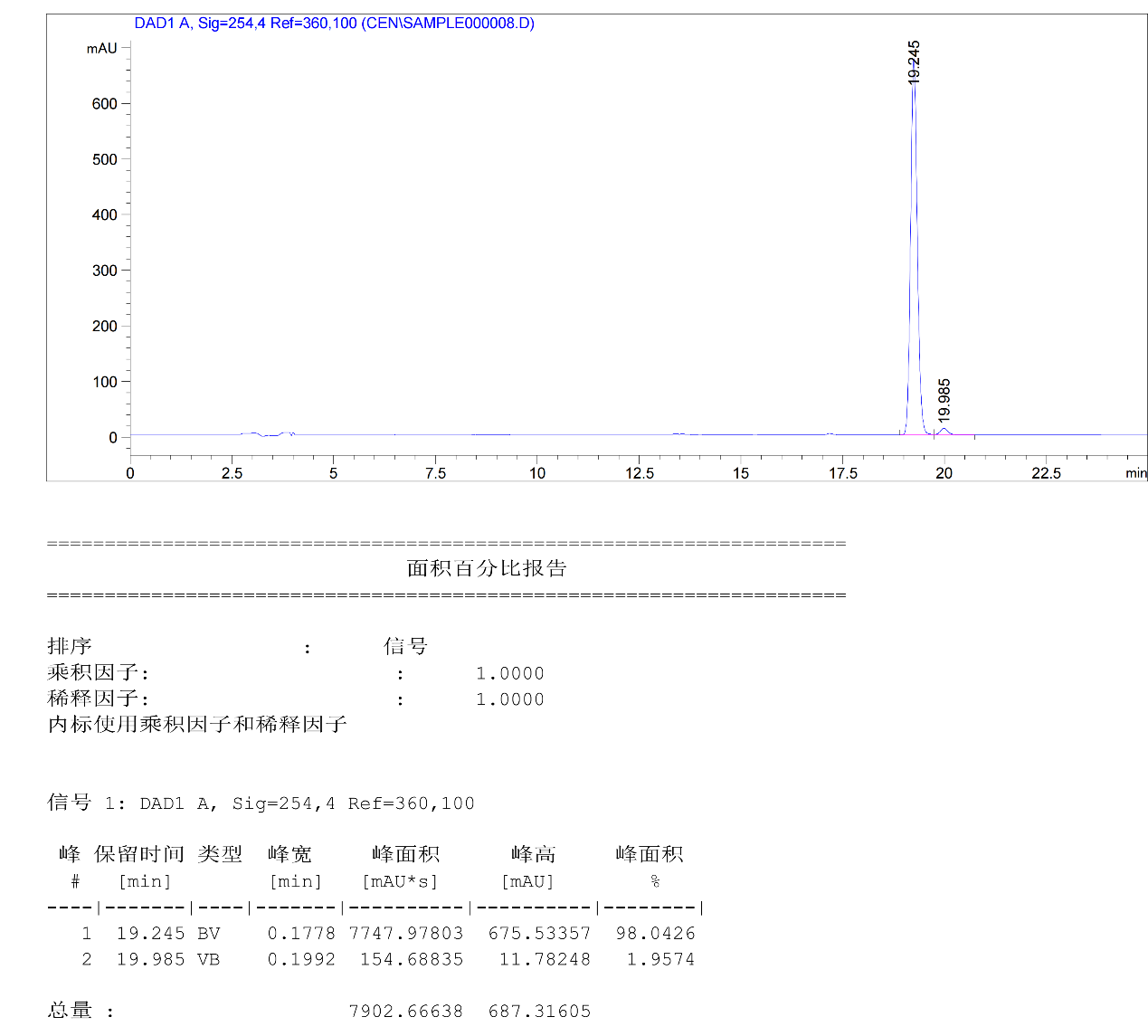


**Figure S6.** HPLC purity analysis of AA-P (>98%).


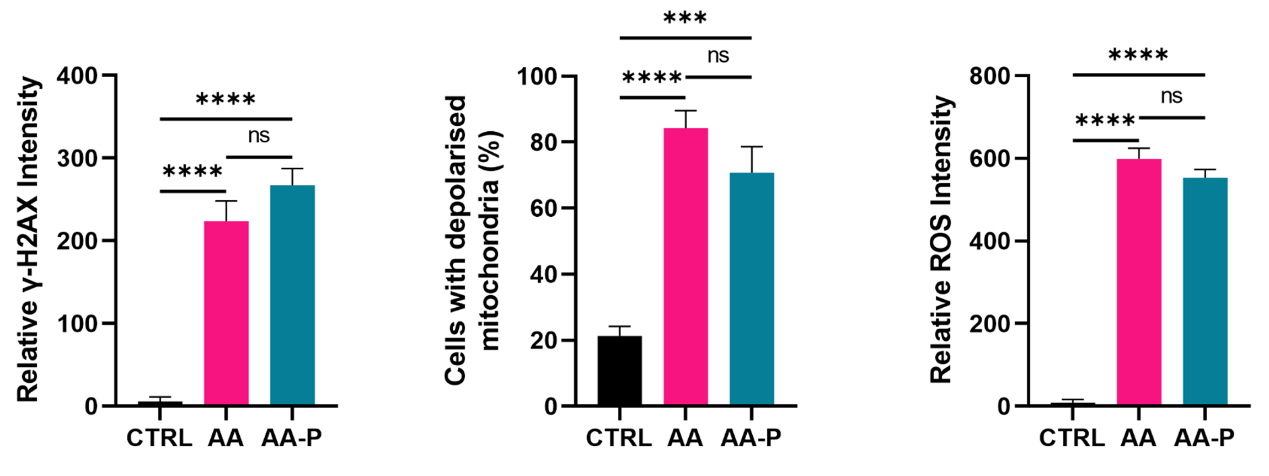


**Figure S7.** Quantification of the fluorescence intensities of ROS production, mitochondrial depolarization, and γ-H2A expression in HK-2 cells. Student's t-test is used to compare two groups of data affected by a single variable. For the comparison of multiple groups of data, one-way ANOVA is adopted, and Dunnett's multiple comparisons test is used for post hoc analysis. All data are presented as mean ± standard deviation (n = 3). Differences were considered statistically significant at **p* < 0.05, ***p* < 0.01, ****p* < 0.001, and *****p* < 0.0001. NS, not significant.

**Figure S8.** Total ion chromatogram and base peak of the DSMO group (**fraction 1 for the first time**).

**Figure S9.** Total ion chromatogram and base peak of the DSMO group (**fraction 1 for the second time**).

**Figure S10.** Total ion chromatogram and base peak of the DSMO group (**fraction 1 for the third time**).

**Figure S11.** Total ion chromatogram and base peak of the DSMO group (**fraction 2 for the first time**).

**Figure S12.** Total ion chromatogram and base peak of the DSMO group (**fraction 2 for the second time**).

**Figure S13.** Total ion chromatogram and base peak of the DSMO group (**fraction 2 for the third time**).

**Figure S14.** Total ion chromatogram and base peak of the AA-P group (**fraction 1 for the first time**).

**Figure S15.** Total ion chromatogram and base peak of the AA-P group (**fraction 1 for the second time**).

**Figure S16.** Total ion chromatogram and base peak of the AA-P group (**fraction 1 for the third time**).

**Figure S17.** Total ion chromatogram and base peak of the AA-P group (**fraction 2 for the first time**).

**Figure S18.** Total ion chromatogram and base peak of the AA-P group (**fraction 2 for the second time**).

**Figure S19.** Total ion chromatogram and base peak of the AA-P group (**fraction 2 for the third time**).

**Figure S20.** Total ion chromatogram and base peak of the AA-P+AA group (**fraction 1 for the first time**).

**Figure S21.** Total ion chromatogram and base peak of the AA-P+AA group (**fraction 1 for the second time**).

**Figure S22.** Total ion chromatogram and base peak of the AA-P+AA group (**fraction 1 for the third time**).

**Figure S23.** Total ion chromatogram and base peak of the AA-P+AA group (**fraction 2 for the first time**).

**Figure S24.** Total ion chromatogram and base peak of the AA-P+AA group (**fraction 2 for the second time**).

**Figure S25.** Total ion chromatogram and base peak of the AA-P+AA group (**fraction 2 for the third time**).


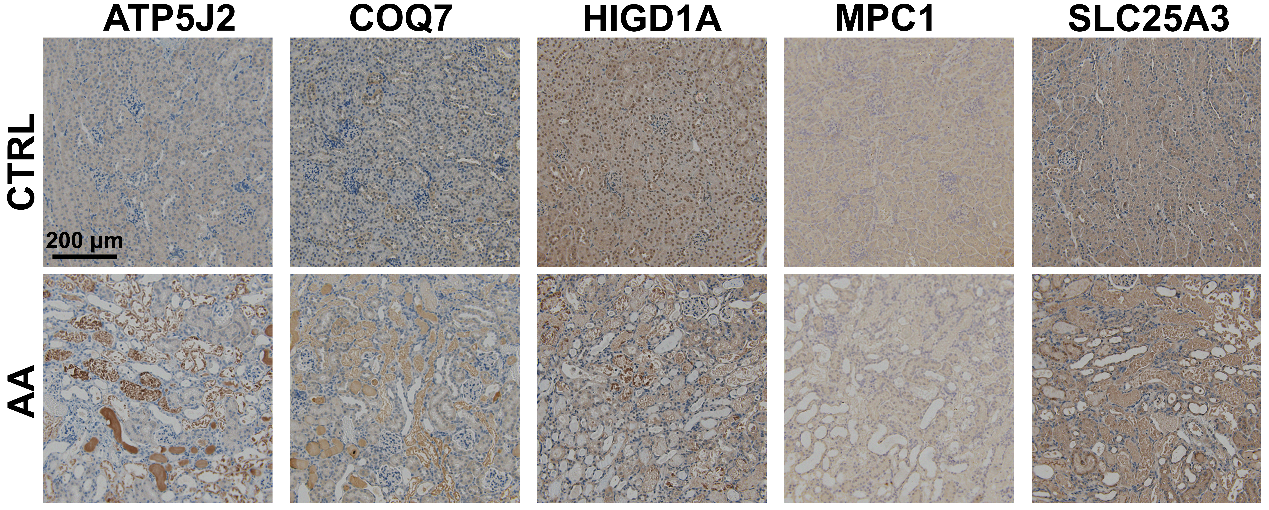


**Figure S26.** ATP5J2, COQ7, HIGD1A, MPC1, or SLC25A3 expression in mouse kidneys was measured via an IHC assay.


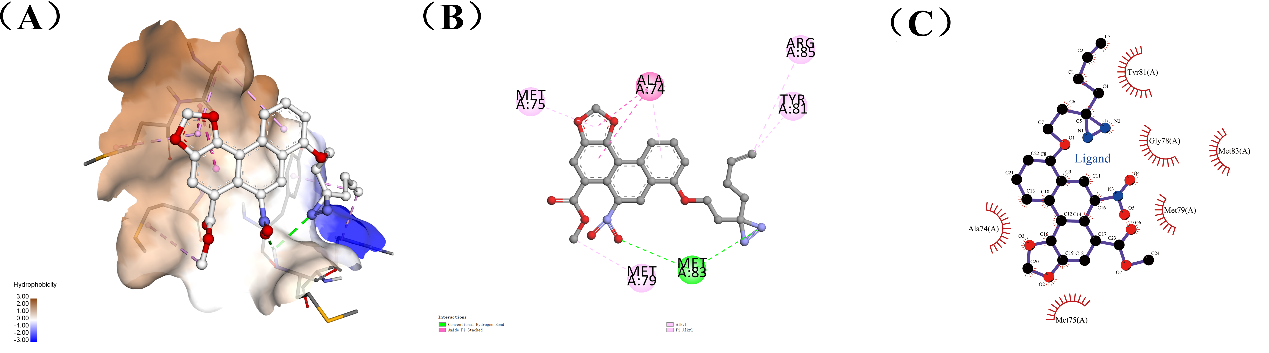


**Figure S27.** (A) 3D hydrophobic interaction mapping. (B) 2D diagram of protein-ligand interaction network. (C) 2D schematic of hydrophobic contacts.


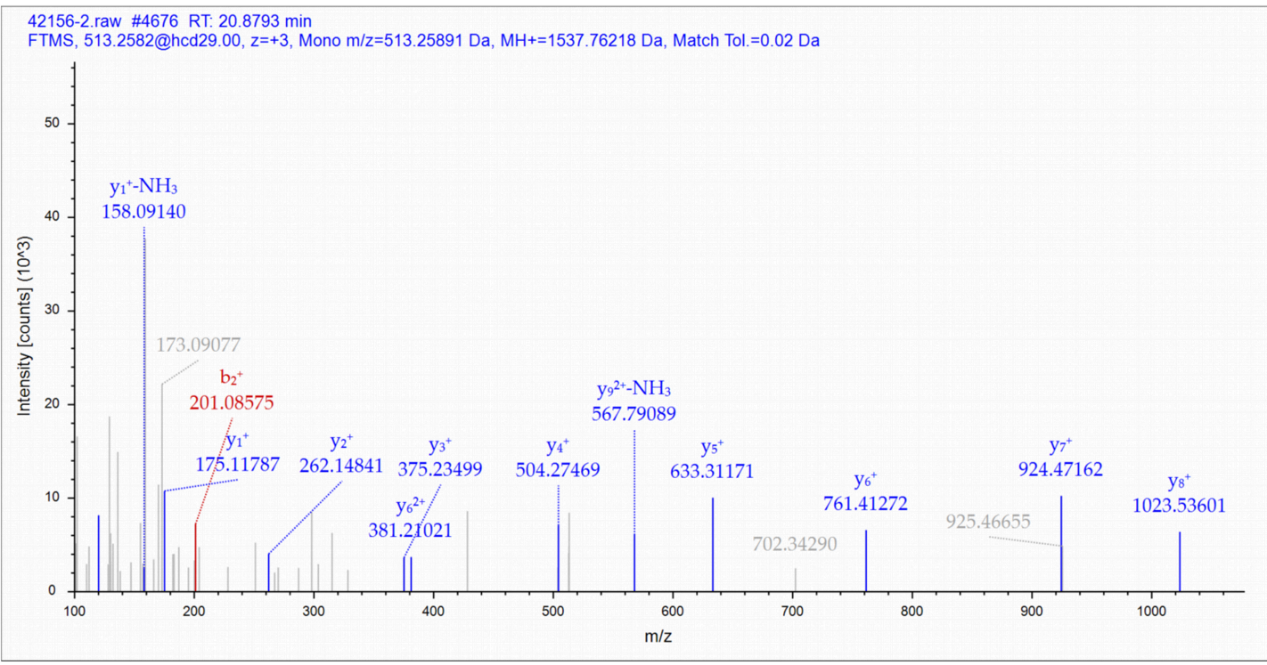


**Figure S28.** LC-MS/MS analysis showed that HIGD1A binds to TFAM.


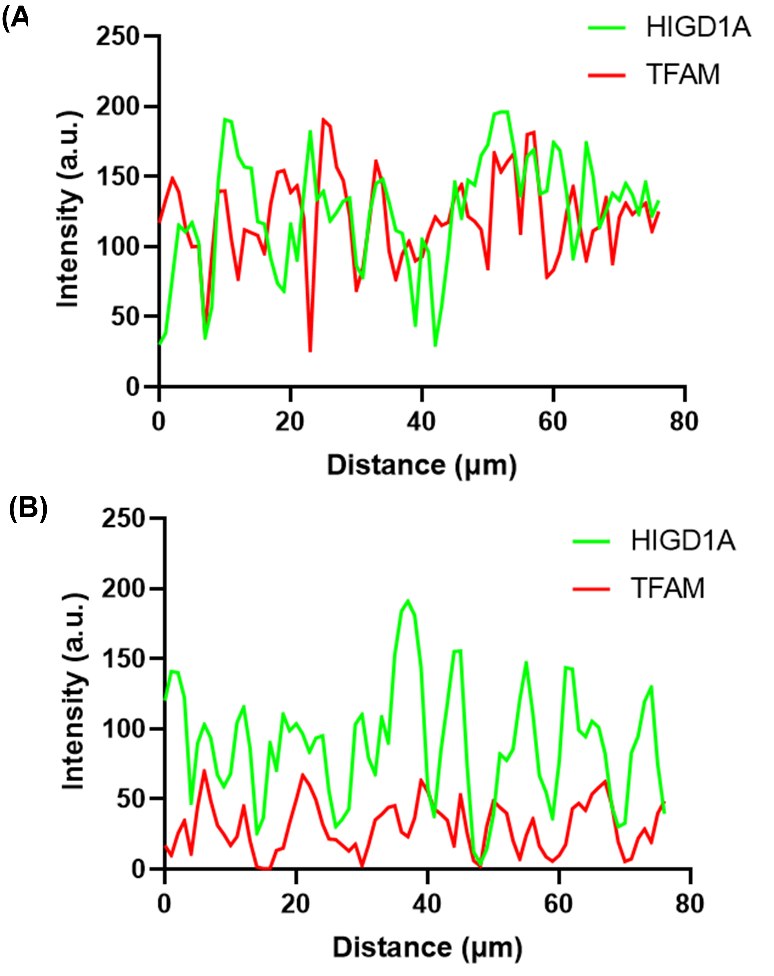


**Figure S29.** Co-localization of HIGD1A (green) and TFAM (red) by immunofluores-cence analysis.


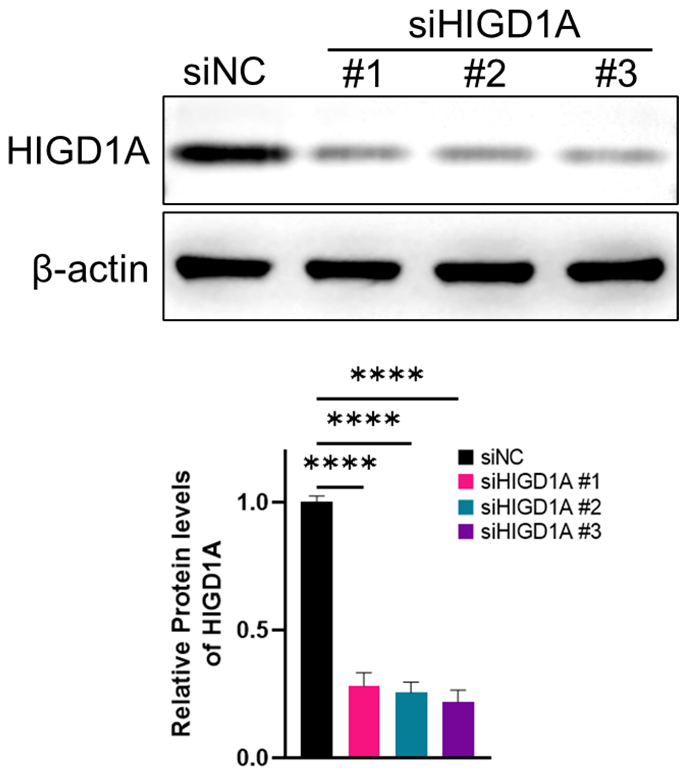


**Figure S30.** Western blot and quantitative analysis of HIGD1A protein was performed on HK-2 cells transfected with si-RNA.


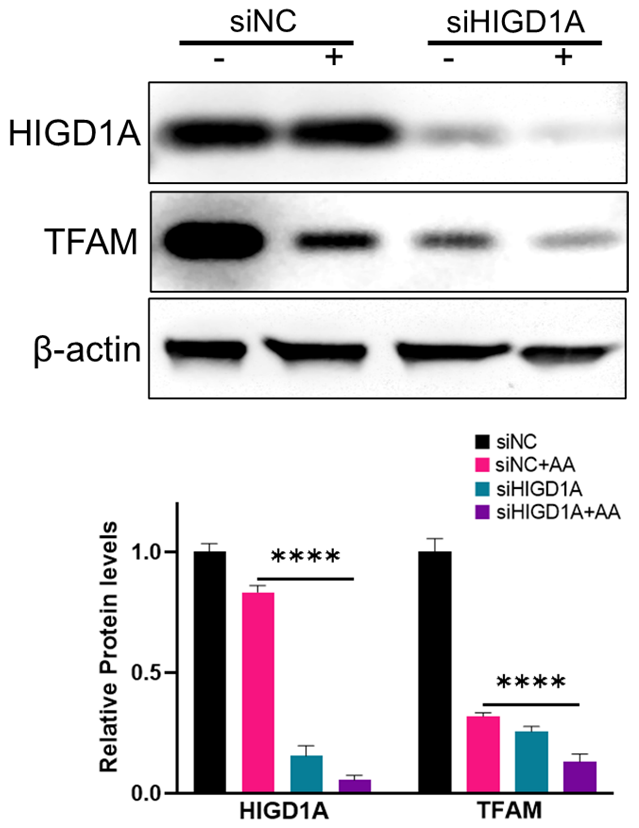


**Figure S31.** Western blot and quantitative analysis of HIGD1A and TFAM proteins were performed on siRNA-transfected HK-2 cells (20 μM si-NC or si-HIGD1A for 24 h).


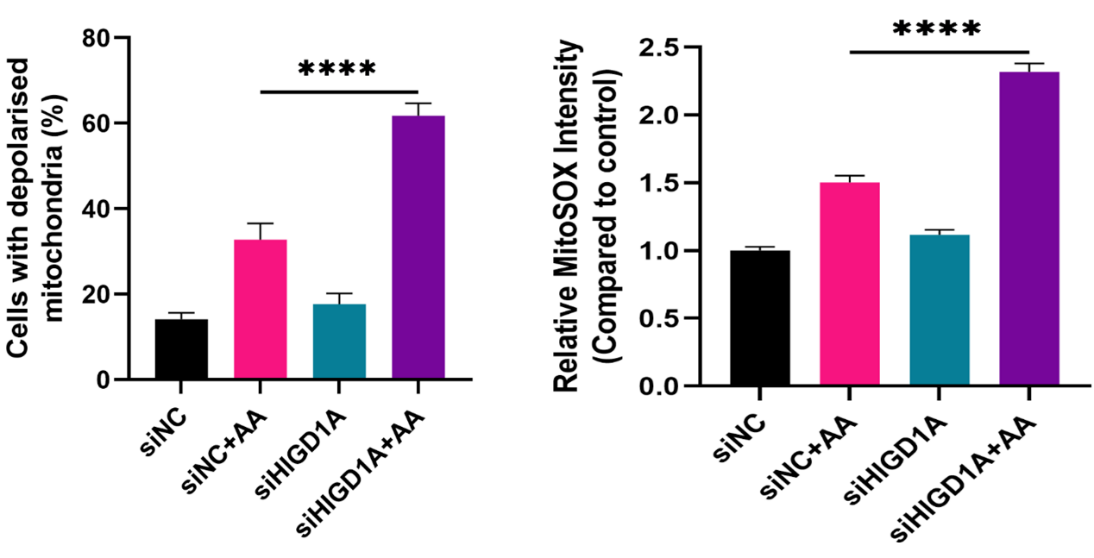


**Figure S32.** Quantification of the fluorescence intensities of ROS production, mitochondrial depolarization, and γ-H2A expression in HK-2 cells. n = 3 per group. Student's t-test is used to compare two groups of data affected by a single variable. All data are presented as mean ± standard deviation. Differences were considered statistically significant at **p* < 0.05, ***p* < 0.01, ****p* < 0.001, and *****p* < 0.0001.


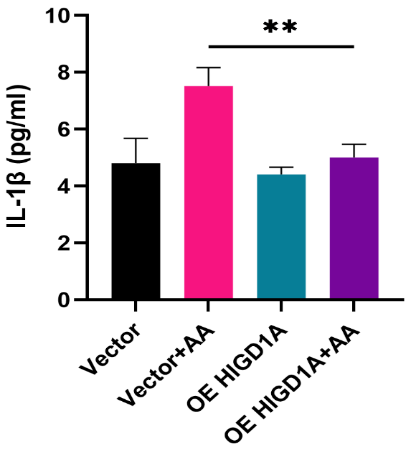


**Figure S33.** The concentrations of IL-1β were measured by ELISA. n = 3 per group. Student's t-test is used to compare two groups of data affected by a single variable. All data are presented as mean ± standard deviation. Differences were considered statistically significant at **p* < 0.05, ***p* < 0.01, ****p* < 0.001, and *****p* < 0.0001.


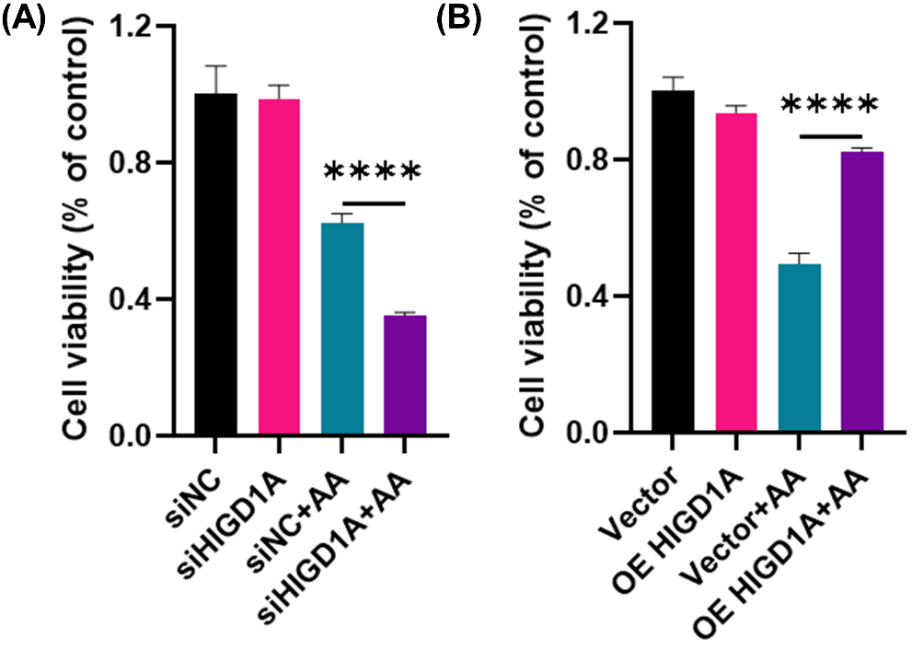


**Figure S34.** Effects of HIGD1A knockdown(A) and overexpression(B) on the viability of AA-treated HEK293T cells. n = 3 per group. Student's t-test is used to compare two groups of data affected by a single variable. All data are presented as mean ± standard deviation. Differences were considered statistically significant at **p* < 0.05, ***p* < 0.01, ****p* < 0.001, and *****p* < 0.0001.


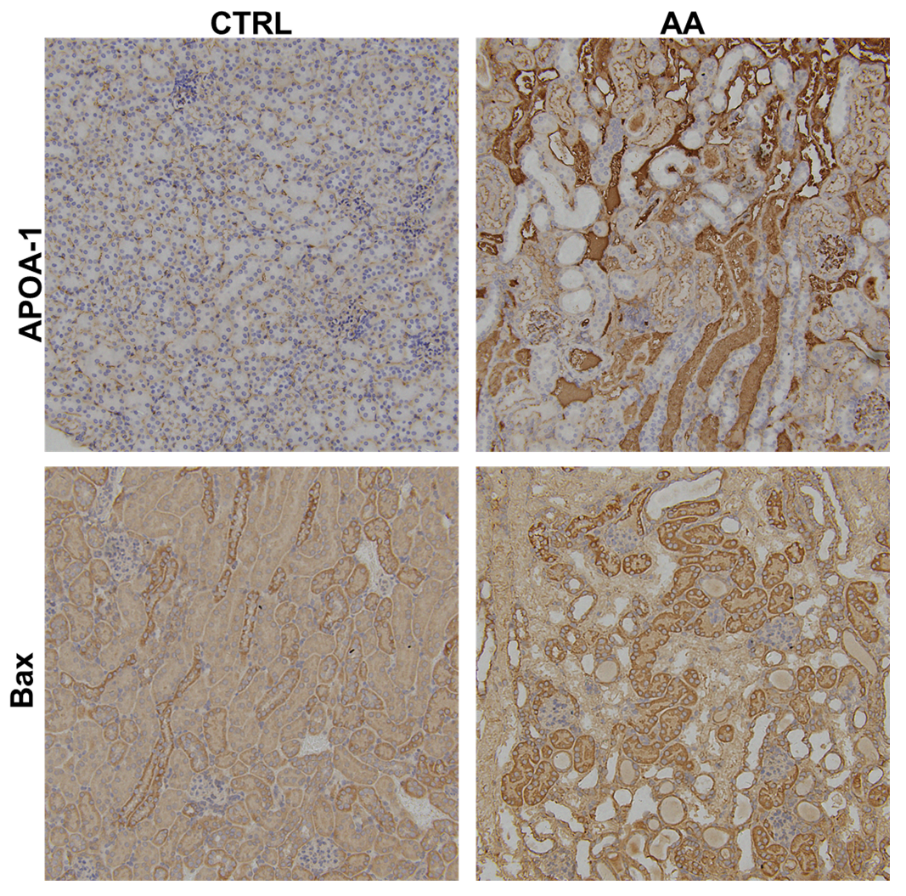


**Figure S35.** APOA-1 and Bax expression in mouse kidneys as detected by an IHC assay.

**Table S1** Primers for genes in RT-PCR

| **Gene** | **Sense（5’-3’）** | **Anti-Sense（3’-5’）** |
| --- | --- | --- |
| *JUN* | GCCAAGAACTCGGACCTCCTC | GCCTGGGTTGAAGTTGCTGAG |
| *FOS* | GCCTTCACCCTGCCTCTCCTC | TCATTGCTGCTGCTGCCCTTG |
| *FOSB* | ACAACCAGCCAGGACCTCCAG | TCCAACTGATCTGTCTCCGTCTCC |
| *ATF3* | TTGCTAACCTGACGCCCTTTGTC | CACACTTTCCAGCTTCTCCGACTC |
| *IL-6* | ACTCACCTCTTCAGAACGAATTG | CCATCTTTGGAAGGTTCAGGTTG |
| *TNF-α* | CCTCTCTCTAATCAGCCCTCTG | GAGGACCTGGGAGTAGATGAG |
| *IL-1β* | ATGATGGCTTATTACAGTGGCAA | GTCGGAGATTCGTAGCTGGA |
| *IL-10* | GTTACCTGGGTTG | TCACATGCGCCTTGATGTCTG |
| *ATP5MF* | GGCGTCAGTTGTACCAGTGAAGG | GCTCGTGCTTGAGATGCTTGTAGG |
| *MPC1* | CGGATGACATTTGCCTCGGA | TGGCAATGCTGTCCCTTCAA |
| *HIGD1A* | GAGCAGAAGCTTGTGGAGGAGATTC | ACGCATGTGGATCAGATGAATGGAC |
| *COQ7* | GGTGGATCATGCAGGCGAATATGG | TGTCATGGTGCTCAAGCTCTTCATC |
| *SLC25A3* | CGCCGTGGAAGAGTACAGTTGTG | GGAGCCCATCCTTTAGCCAAACC |
| *CYTB* | GCC TGC CTG ATC CTC CAA AT | AAG GTA GCG GAT GAT TCA GCC |
| *TERT* | CTAGCTCATGTGTCAAGACCCTCTT | GCCAGC ACGTTTCTCTCGTT |
| *D-LOOP* | CTAAATAGCCCACACGTTCC | TAGGATGAGGCAGGAATCAA |
| *JUN*=jun proto-oncogene; *FOS*=fos proto-oncogene; *FOSB*=fosb proto-oncogene;  *ATF3*=activating transcription factor 3; *IL-6*=interleukin-6; *TNF-α*=tumor necrosis factor- α;  *IL-1β*=interleukin-1β; *IL-10*=interleukin-6; *CYTB* =mitochondrially Encoded Cytochrome B;  *TERT* =telomerase Reverse Transcriptase; *D-LOOP=*mitochondrial displacement loop | | |

**Table S2** siRNA sequences for the targeted genes

| **Gene** | **Sense（5’-3’）** | **Anti-Sense（3’-5’）** |
| --- | --- | --- |
| *siATP5MF-170* | CUACCGGUACUACAACAAGTT | CUUGUUGUAGUACCGGUAGTT |
| *siATP5MF-40* | GUGAGUGUCCGGCCCCAGUTT | ACUGGGGCCGGACACUCACTT |
| *siATP5MF-291* | CGGCUCCGCAAAUACCACUTT | AGUGGUAUUUGCGGAGCCGTT |
| *siMPC1-311* | CCACGCAACAAAUGAAGUATT | UACUUCAUUUGUUGCGUGGTT |
| *siMPC1-224* | GAUGACAUUUGCCCUCUGUTT | ACAGAGGGCAAAUGUCAUCTT |
| *siMPC1-95* | CUAUGUCCGAAGCAAGGAUTT | AUCCUUGCUUCGGACAUAGTT |
| *siMPC1-236* | GCCCUCUGUUGCUAUUCUUTT | AAGAAUAGCAACAGAGGGCTT |
| *siHIGD1A-245* | CCAUUCAUCUGAUCCACAUTT | AUGUGGAUCAGAUGAAUGGTT |
| *siHIGD1A-104* | CUUCAUAUGAGGAAGAUCATT | UGAUCUUCCUCAUAUGAAGTT |
| *siCOQ7-666* | GAUGCAGAGUGGCGAUAUATT | UAUAUCGCCACUCUGCAUCTT |
| *siCOQ7-222* | CAGGCGAAUAUGGAGCAAATT | UUUGCUCCAUAUUCGCCUGTT |
| *siCOQ7-480* | GCAUAGCACAUCACUACAATT | UUGUAGUGAUGUGCUAUGCTT |
| *siCOQ7-139* | CAGUGUCAGAUUUCGCAGUTT | ACUGCGAAAUCUGACACUGTT |
| *siSLC25A3-531* | CUGGCGCACAUCACUAUAUTT | AUAUAGUGAUGUGCGCCAGTT |
| *siSLC25A3-840* | GCUGGUUGUAACAUUUGUATT | UACAAAUGUUACAACCAGCTT |
| *siSLC25A3-358* | GGCAUAUUUAACGGAUUCUTT | AGAAUCCGUUAAAUAUGCCTT |
| *siSLC25A3-963* | CAAGAGACUUGGAUUUAAATT | UUUAAAUCCAAGUCUCUUGTT |
| *siCTRL* | UUCUCCGAACGUGUCACGUTT | ACGUGACACGUUCGGAGAATT |
| *si-ATP5MF*= ATP Synthase Membrane Subunit F; *si-MPC1*=Mitochondrial Pyruvate Carrier 1;  *si-HIGD1A*= HIG1 Hypoxia Inducible Domain Family Member 1A; *si-COQ7*=Coenzyme Q7;  *si-SLC25A3*=Solute Carrier Family 25 Member 3; *si-NC*=negative control | | |

**Source Data**


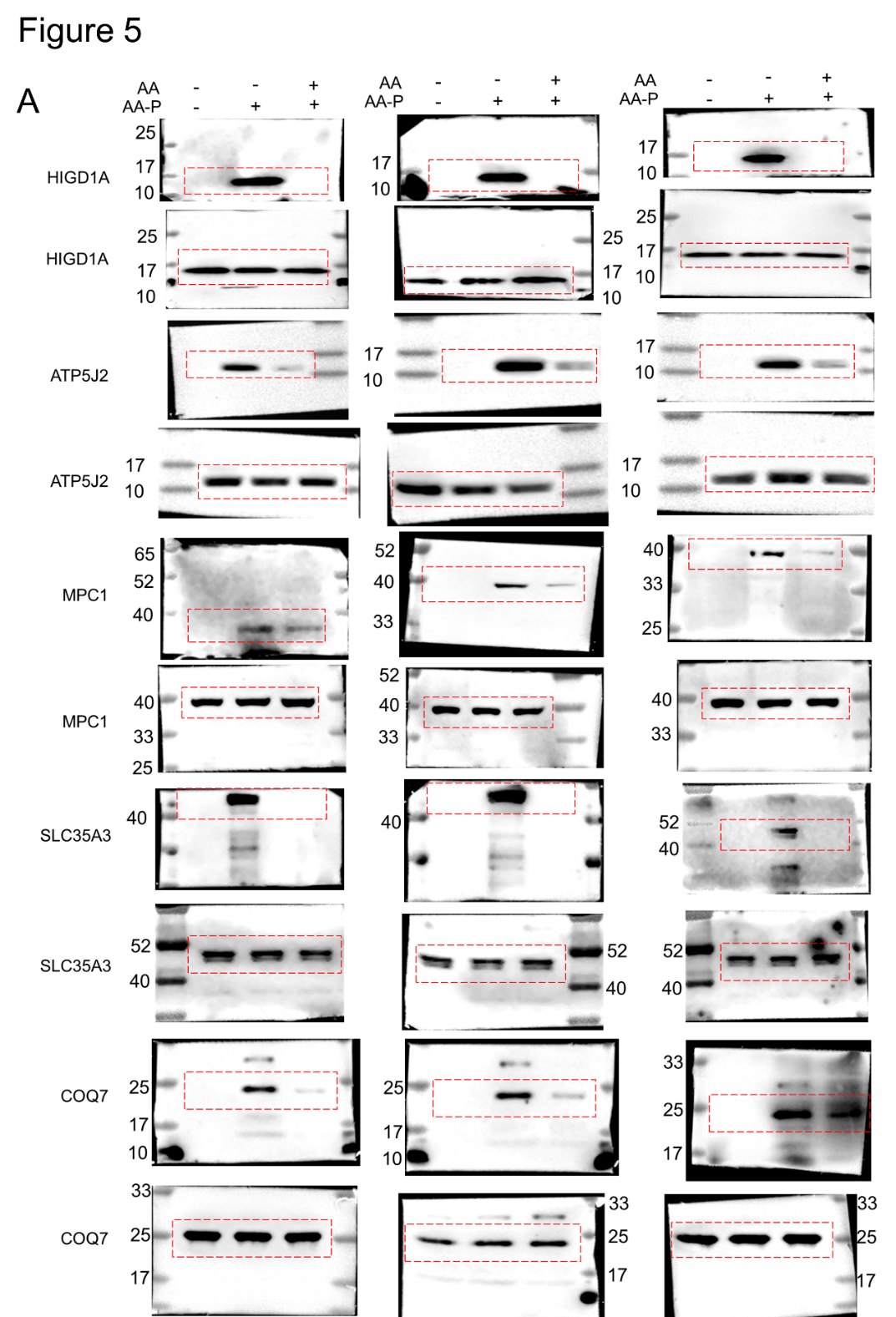


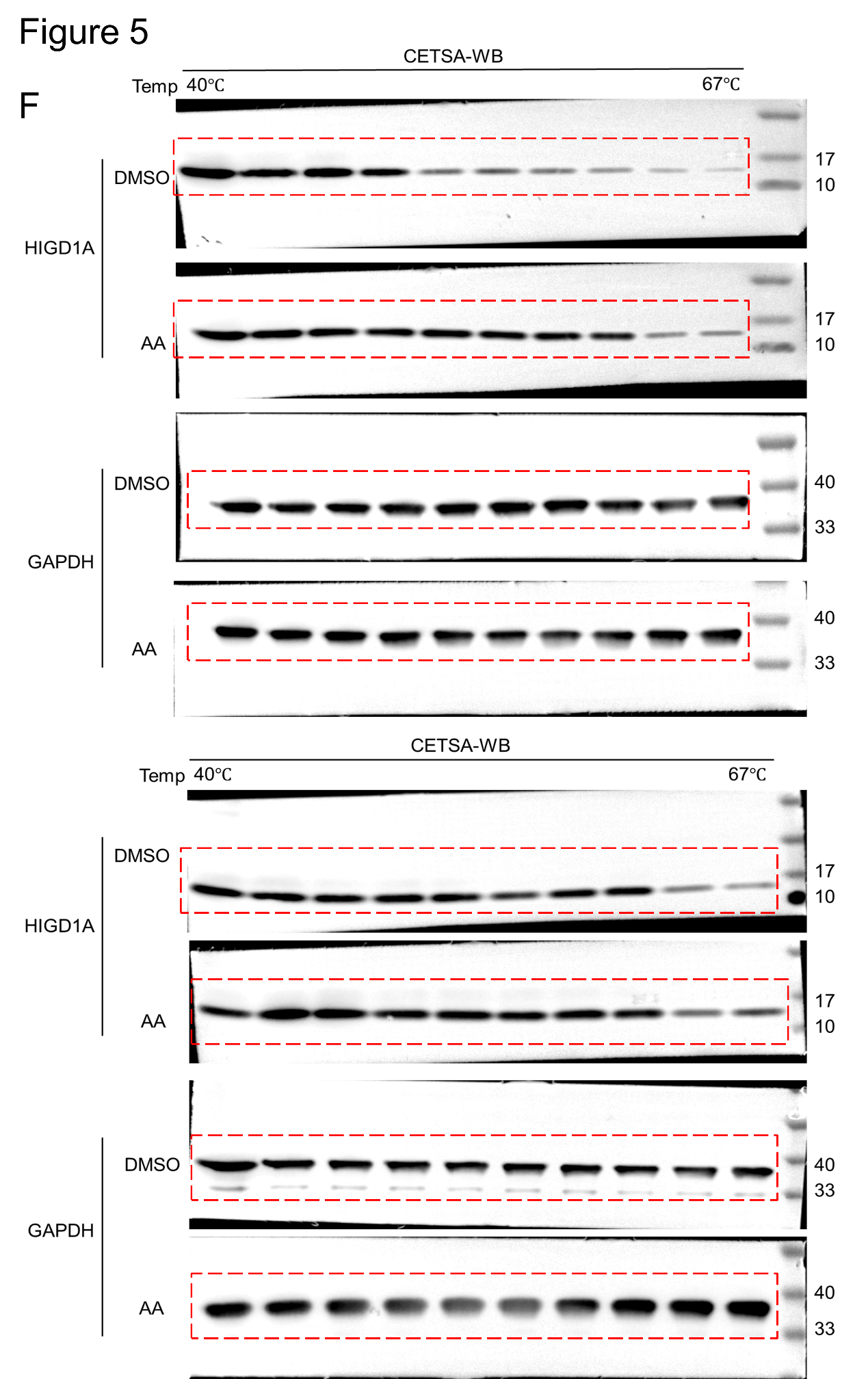


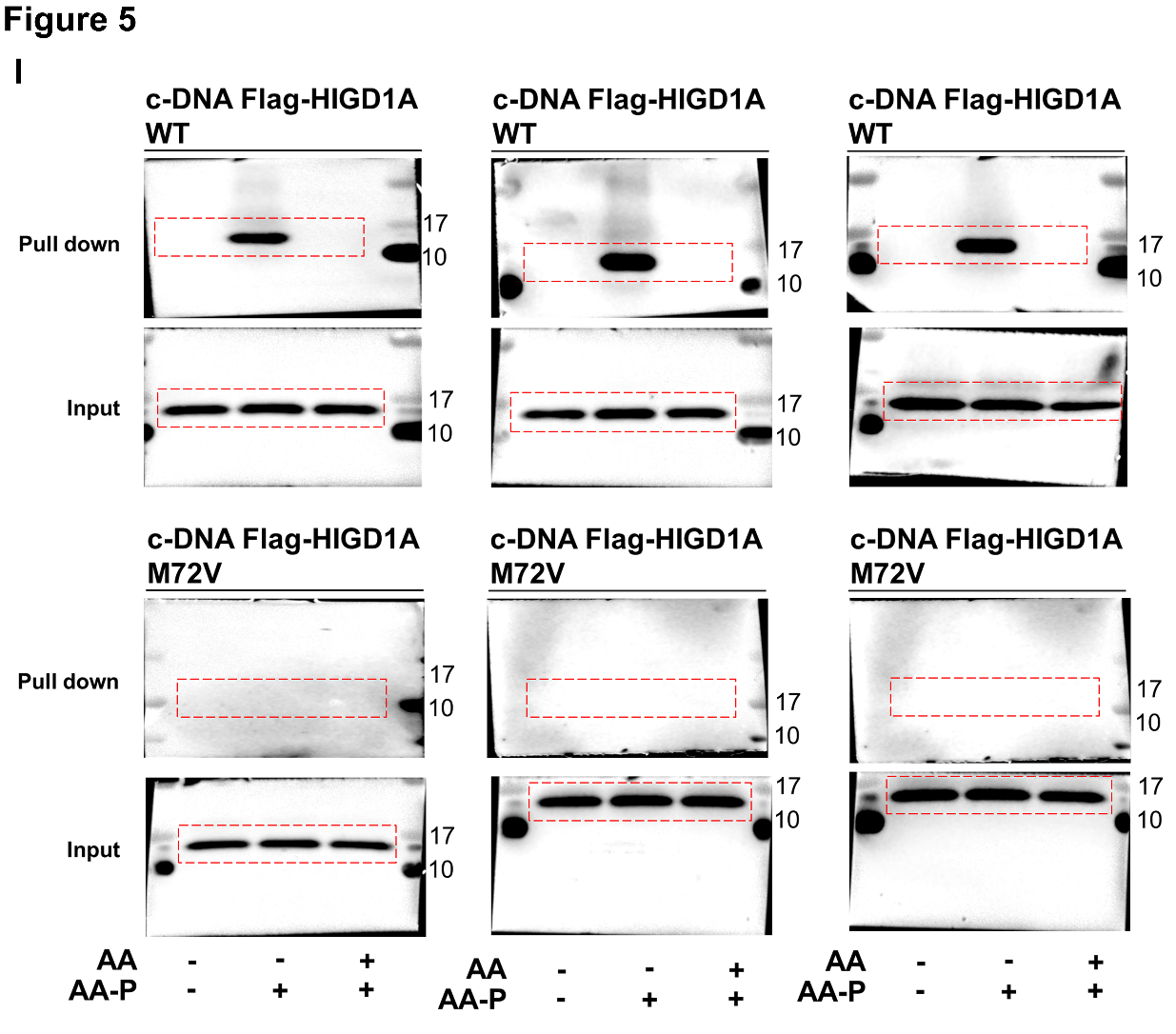


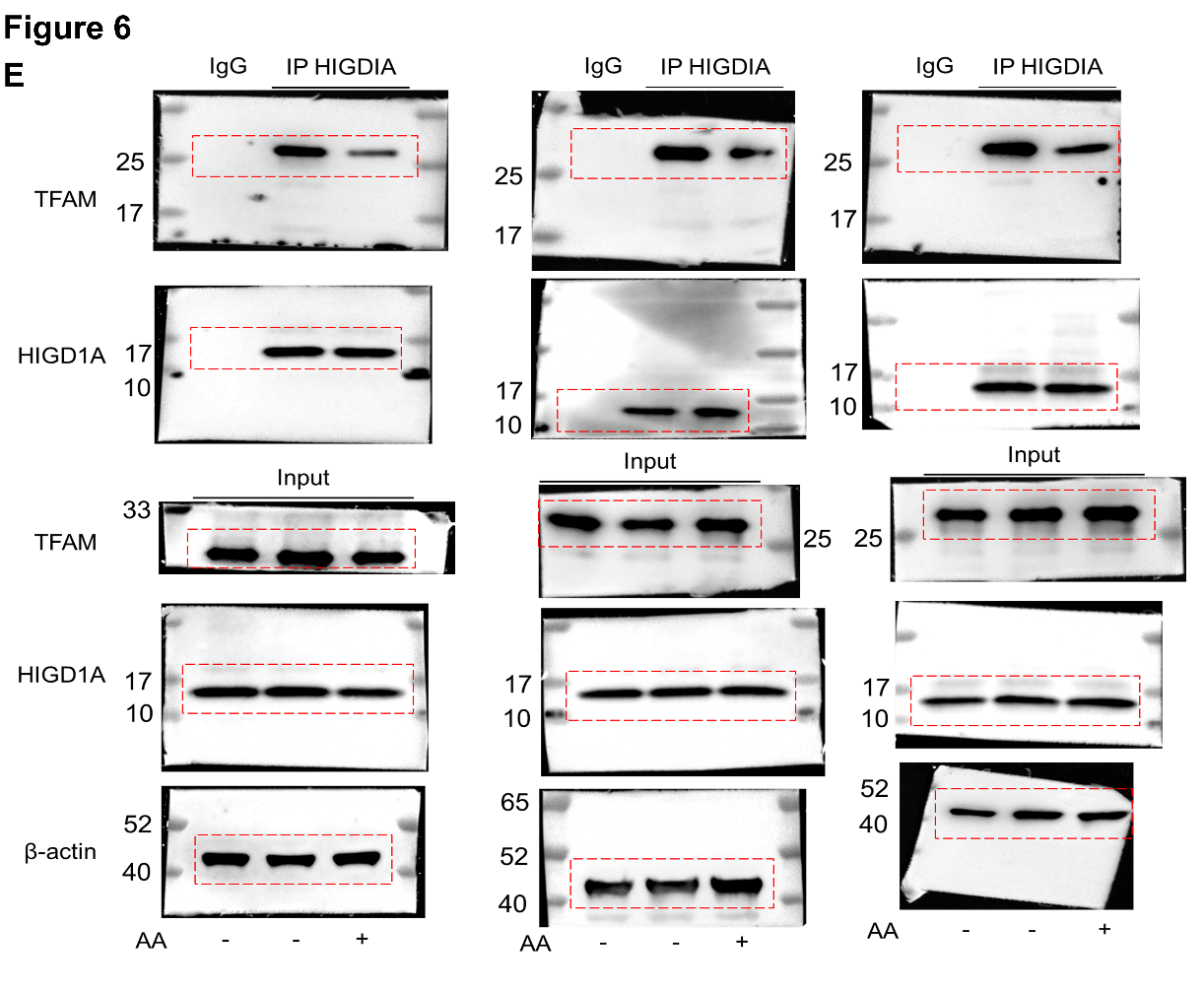


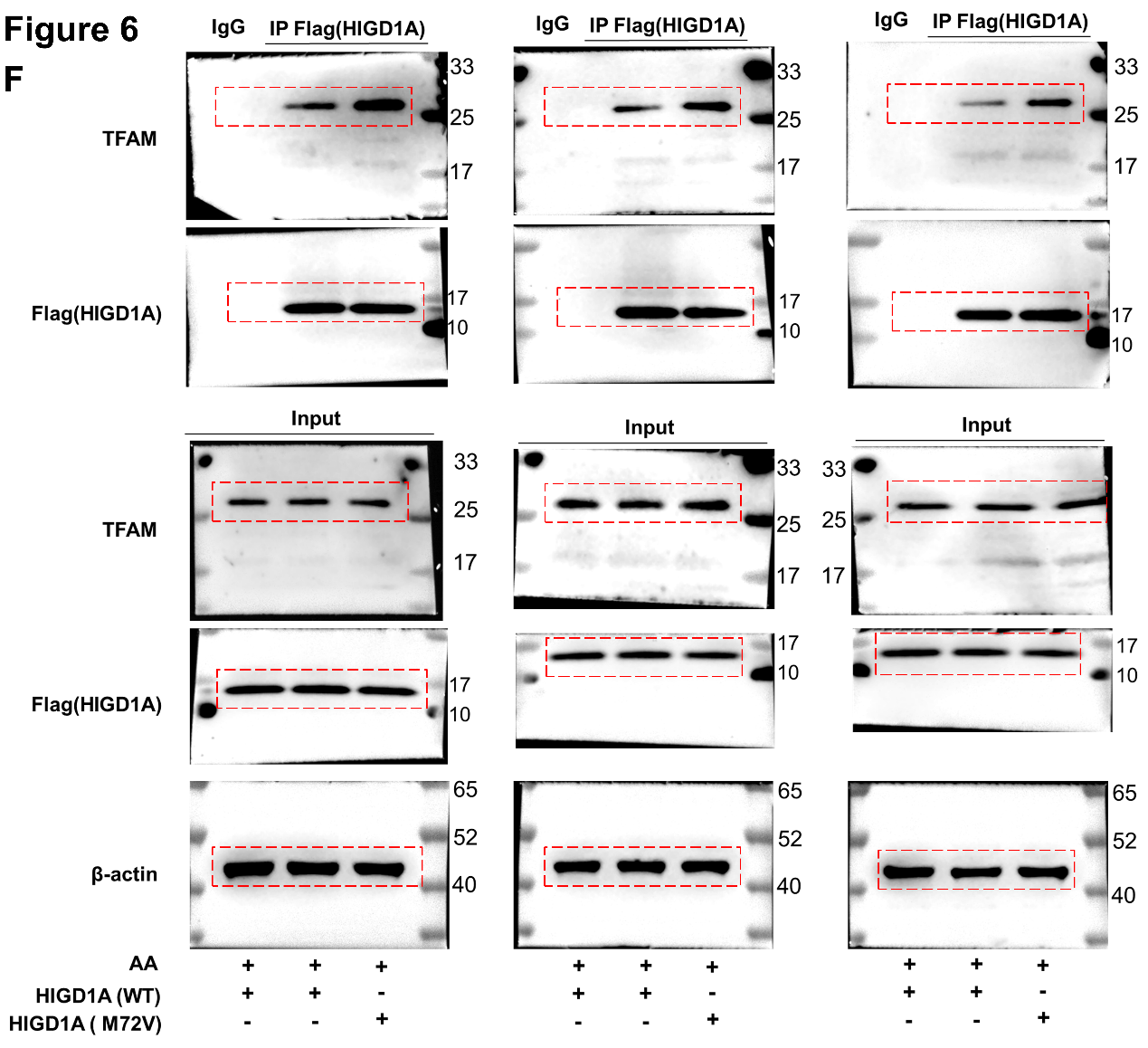


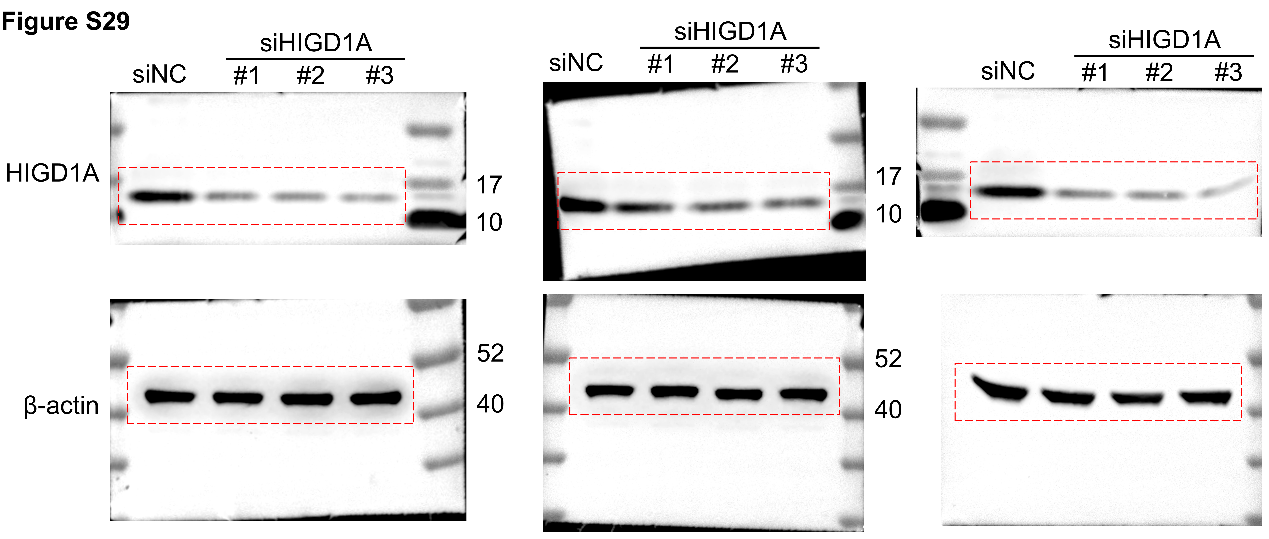


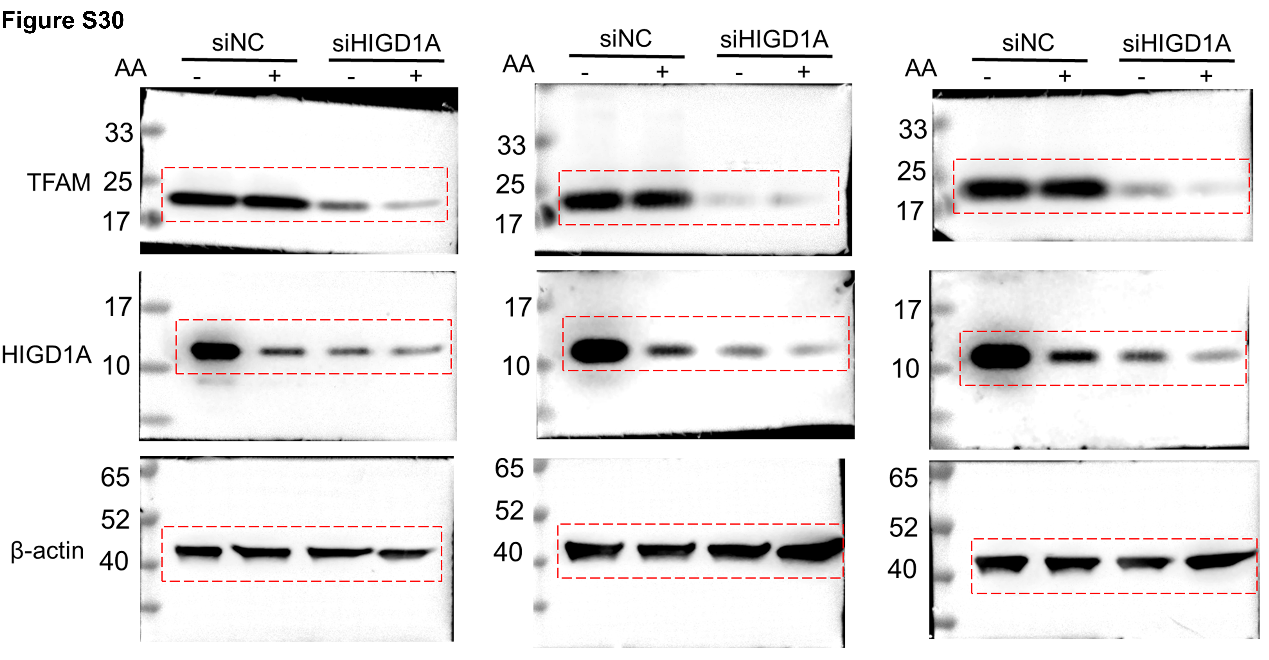


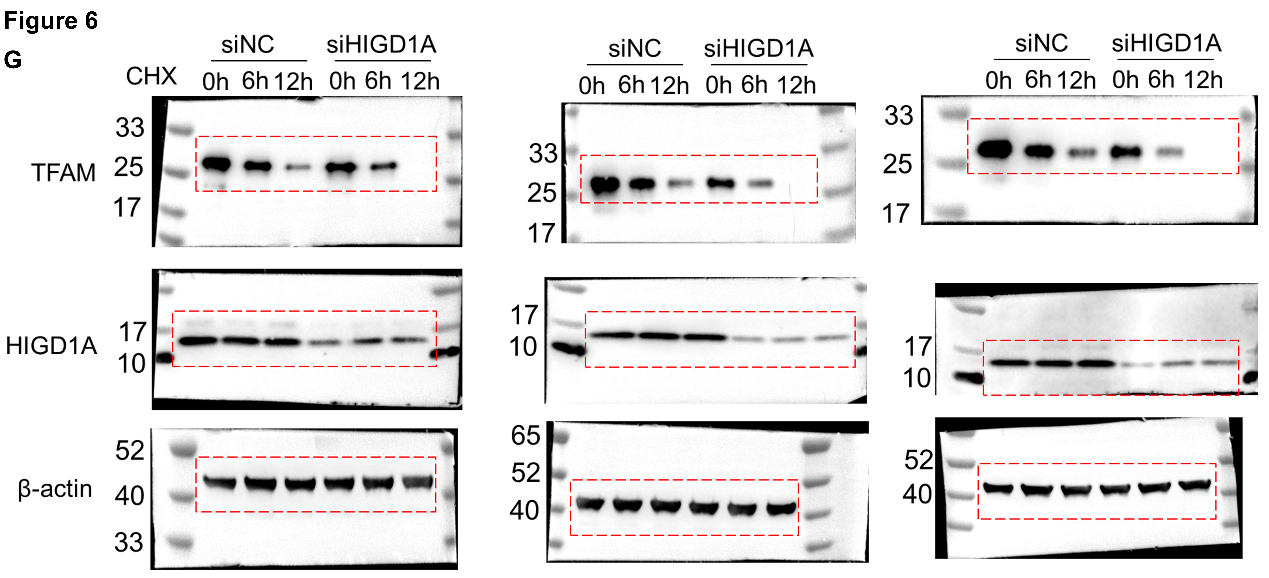


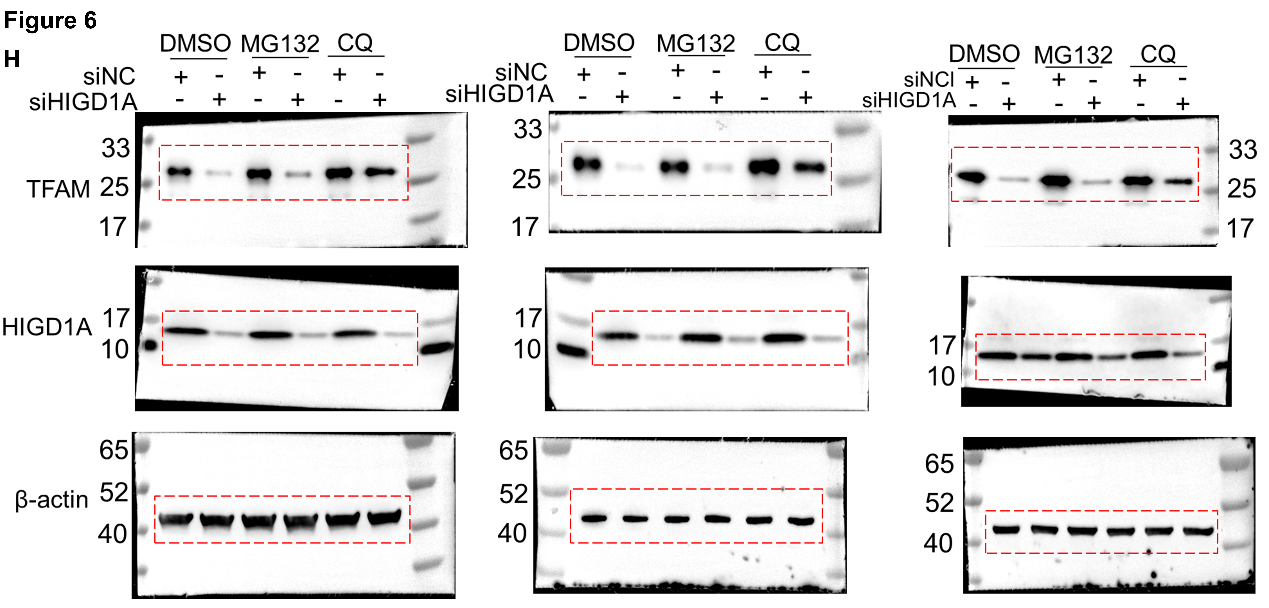


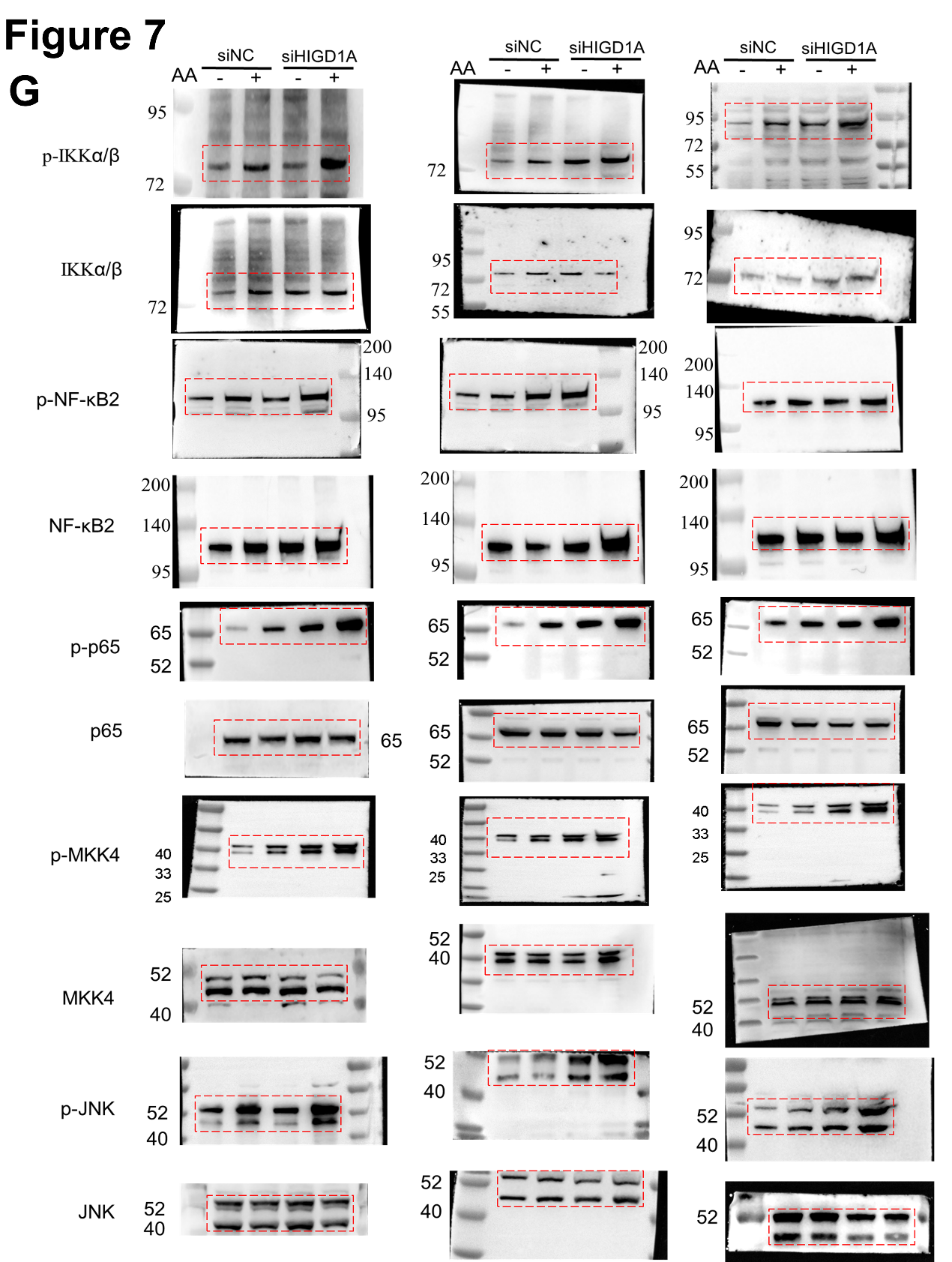


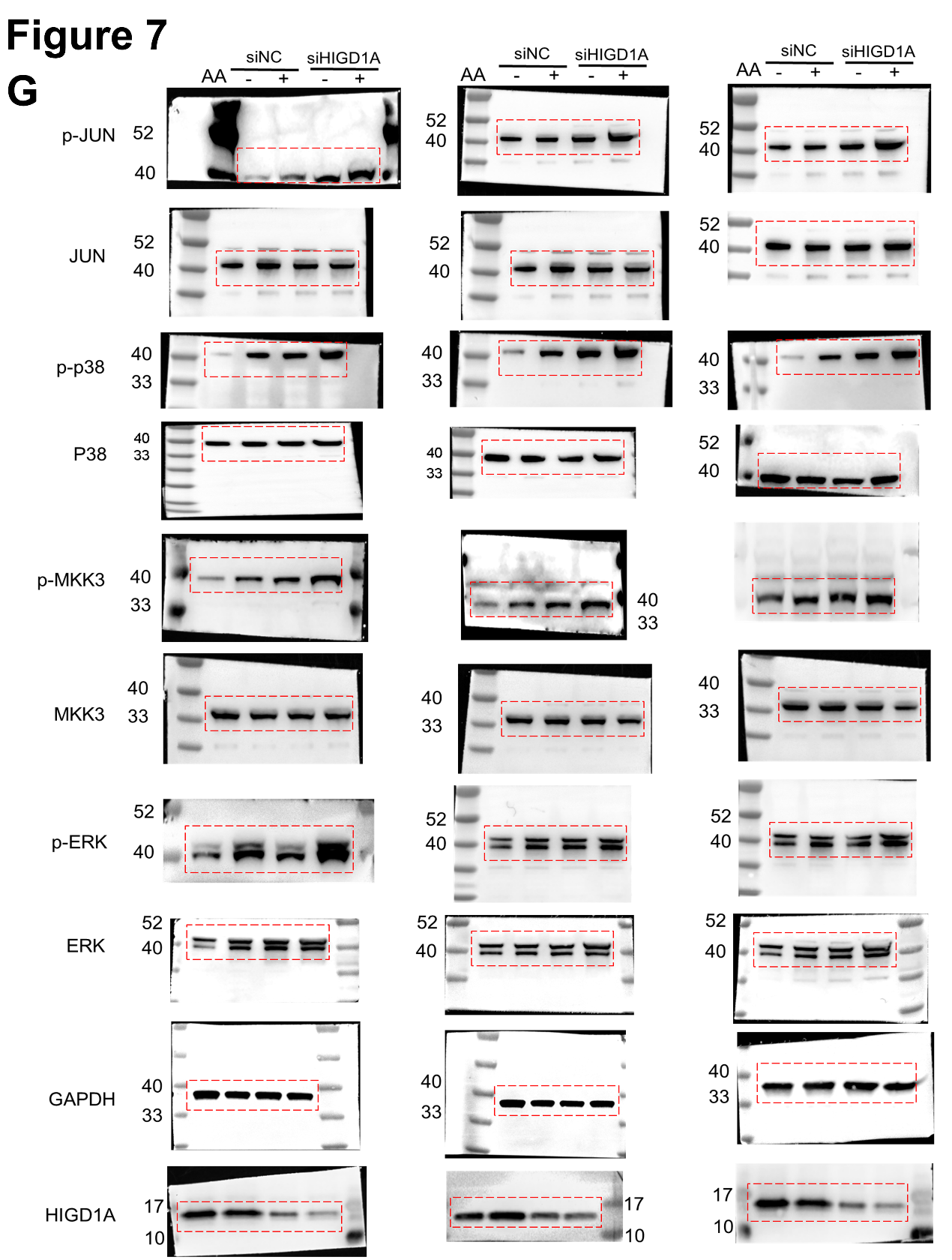


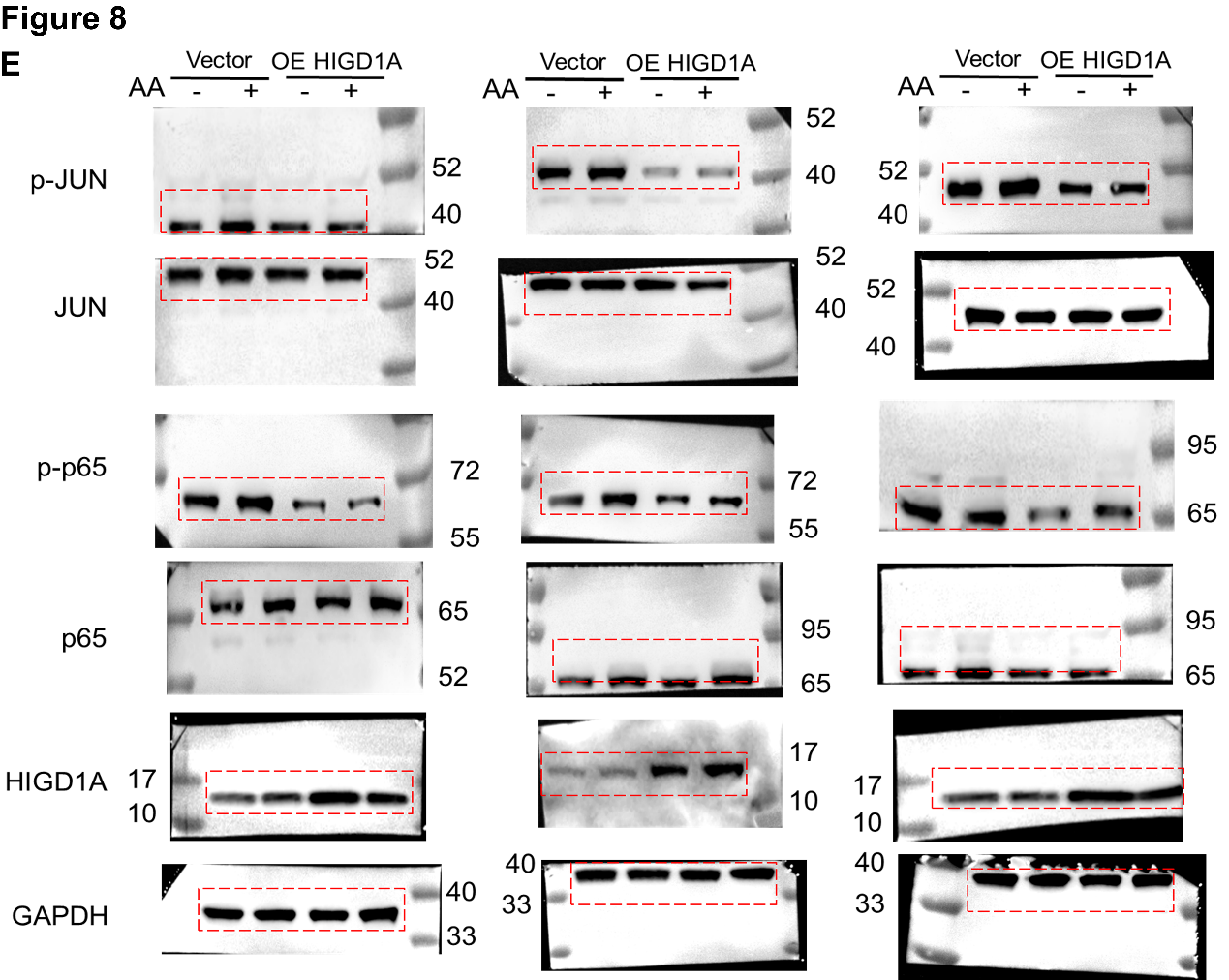

Supplement: Supplementary file 1 — Supporting Information [file ADVS-13-e13117-s001.docx]
